# HAT1 signaling confers to assembly and epigenetic regulation of HBV cccDNA minichromosome

**DOI:** 10.7150/thno.37173

**Published:** 2019-09-25

**Authors:** Guang Yang, Jinyan Feng, Yunxia Liu, Man Zhao, Ying Yuan, Hongfeng Yuan, Haolin Yun, Mingming Sun, Yanan Bu, Lei Liu, Zixian Liu, Jun-qi Niu, Ming Yin, Xijun Song, Zhenchuan Miao, Zhongqing Lin, Xiaodong Zhang

**Affiliations:** 1 Department of Cancer Research, Institute of Molecular Biology, College of Life Sciences, Nankai University, Tianjin 300071, P.R. China.; 2 Department of Hepatology, The First Hospital, Jilin University, Changchun 130021, P.R. China.; 3 Beijing Vitalstar Biotechnology Co. Ltd., Beijing 100000, P.R. China.

**Keywords:** HAT1 signaling, HBV cccDNA minichromosome, assembly, epigenetic modification

## Abstract

**Rationale**: Hepatitis B virus (HBV) is a leading cause of liver diseases. HBV covalently closed circular DNA (cccDNA) is a critical obstacle of complete elimination by anti-HBV therapy. HBV cccDNA accumulates in nucleus as a chromatin-like cccDNA minichromosome assembled by histones and non-histones. However, the underlying mechanism of modulation of cccDNA minichromosome in hepatocytes is poorly understood.

**Methods**: A human liver-chimeric mouse model was established. The cccDNA-ChIP, Southern blot analysis, confocal assays, RIP assays and RNA pull-down assays, et al. were performed to assess the mechanism of assembly and epigenetic regulation of cccDNA minichromosome in human liver-chimeric mouse model, human primary hepatocytes (PHH), dHepaRG, HepG2-NTCP cell lines and clinical liver tissues.

**Results**: Importantly, the expression levels of HAT1, CAF-1 and lncRNA HULC were significantly elevated in the liver from HBV-infected human liver-chimeric mice. Strikingly, the depletion of HAT1 reduced HBV replication and cccDNA accumulation, and impaired the assembly of histone H3/H4 and the deposition of HBx and p300 onto cccDNA to form cccDNA minichromosome in the cells. Mechanically, chromatin assembly factor-1 (CAF-1) was involved in the events. Interestingly, HAT1 modified the acetylation of histone H3K27/H4K5/H4K12 on cccDNA minichromosome. Moreover, lncRNA HULC-scaffold HAT1/HULC/HBc complex was responsible for the modification on cccDNA minichromosome. Additionally, HBV activated HAT1 through HBx-co-activated transcriptional factor Sp1 in a positive feedback manner.

**Conclusion**: HAT1 signaling contributes to assembly and epigenetic regulation of HBV cccDNA minichromosome.

## Introduction

Chronic HBV infection is a leading cause of cirrhosis and liver cancer, resulting in estimated 650 000 deaths per year 1-4. Current antiviral therapies rarely achieve a cure because they do not directly target nuclear HBV covalently closed circular DNA (cccDNA), the genomic form that serves as a HBV replication intermediate and viral persistence reservoir 5-9. As host cellular nucleosome assembly, HBV cccDNA accumulates in hepatocyte nuclei as a stable and chromatin-like minichromosome organized by histone and non-histone. The host molecules that modulate the cccDNA minichromosome are potential targets of anti-HBV therapy. Histone H3, H4, H2A and H2B are the most prominent structural species assembled onto cccDNA to form the original cccDNA minichromosome 10. The virally encoded core antigen (HBc) is also a structural component of the HBV cccDNA minichromosome, preferentially binds to HBV double-stranded DNA 10, 11. In addition, some function proteins, such as hepatitis B virus X protein (HBx) and p300, bind to cccDNA minichromosome. The minichromosome structure ensures the stability and integrity of HBV cccDNA 10. Previous study shows that interferon-α and lymphotoxin-β receptor activation can induce degradation of HBV cccDNA by up-regulating APOBEC3A and APOBEC3B, respectively 8, 12. Recently, our group has reported that HBx-elevated MSL2 modulates HBV cccDNA through inducing degradation of APOBEC3 13. However, the host molecules that modulate the assembly of cccDNA minichromosome are not well documented.

As on cellular DNA, HBV cccDNA minichromosome provides numerous options for dynamic epigenetic control of cccDNA transcriptional activity 14. It has been reported that a series enzymes regulate histone methylation of HBV cccDNA minichromosome, including SETDB1, PRMT5, LSD1 and Set1A 15-17. In addition to histones methylation, histones acetylation can also regulate HBV cccDNA transcription. HBx is assembled onto HBV cccDNA minichromosome, involving the recruitment of HBx partners CBP, p300 and PCAF, and required to HBV replication and the development of liver cancer 18. IFN-α represses viral transcription via epigenetic mechanisms involving the chromatin remodeling polycomb repressive complex 2 (PRC2) 19. Furthermore, in chronic hepatitis patients, high viremia correlates with H3 and H4 hyperacetylation 20. However, the host molecules that confer to the histone acetylation of cccDNA minichromosome remain obscured.

Histone acetyltransferase 1 (HAT1) is the sole known example of type B histone acetyltransferase which is responsible for acetylation of newly synthesized histones, and the acetylation pattern on newly synthesized histones is crucial for nucleosome assembly in host cells 21. As a histone acetyltransferase, HAT1 can also acetylate multiple specific histone sites containing H4K5, H4K12, H3K9, H3K18 and H3K27 22. Hence, HAT1 is a key factor for host chromatin assembly rather than just an enzyme 23. In host cells, the acetylation pattern produced by HAT1 of newly synthesized histone H3 and H4 is recognized by histone chaperones, such as CAF-1 and Asf1, to deposit the histones onto replicated DNA and form nucleosome 21, 24, 25. It has been reported that long noncoding RNAs (lncRNAs) have been implicated primarily in transcriptional regulation by modulating the activity of transcription factors and by serving as scaffolds for assembling transcriptional regulators 26. It has been reported that lncRNA HULC serves as scaffold for YB-1 and its downstream gene to modulate the development of liver cancer 27. However, whether those factors are involved in the modulation of assembly, accumulation and epigenetic regulation of HBV cccDNA minichromosome is elusive.

In this study, we are interested in the role of host molecules in the regulation of HBV cccDNA minichromosome. Strikingly, we found that a host nucleosome assembly HAT1 signaling conferred to the assembly and epigenetic regulation of HBV cccDNA minichromosome. Thus, our finding provides new insights into the mechanism by which the host HAT1 signaling modulates episomal DNA viruses.

## Results

### HAT1 contributes to HBV replication and cccDNA accumulation

**Figure 1:**
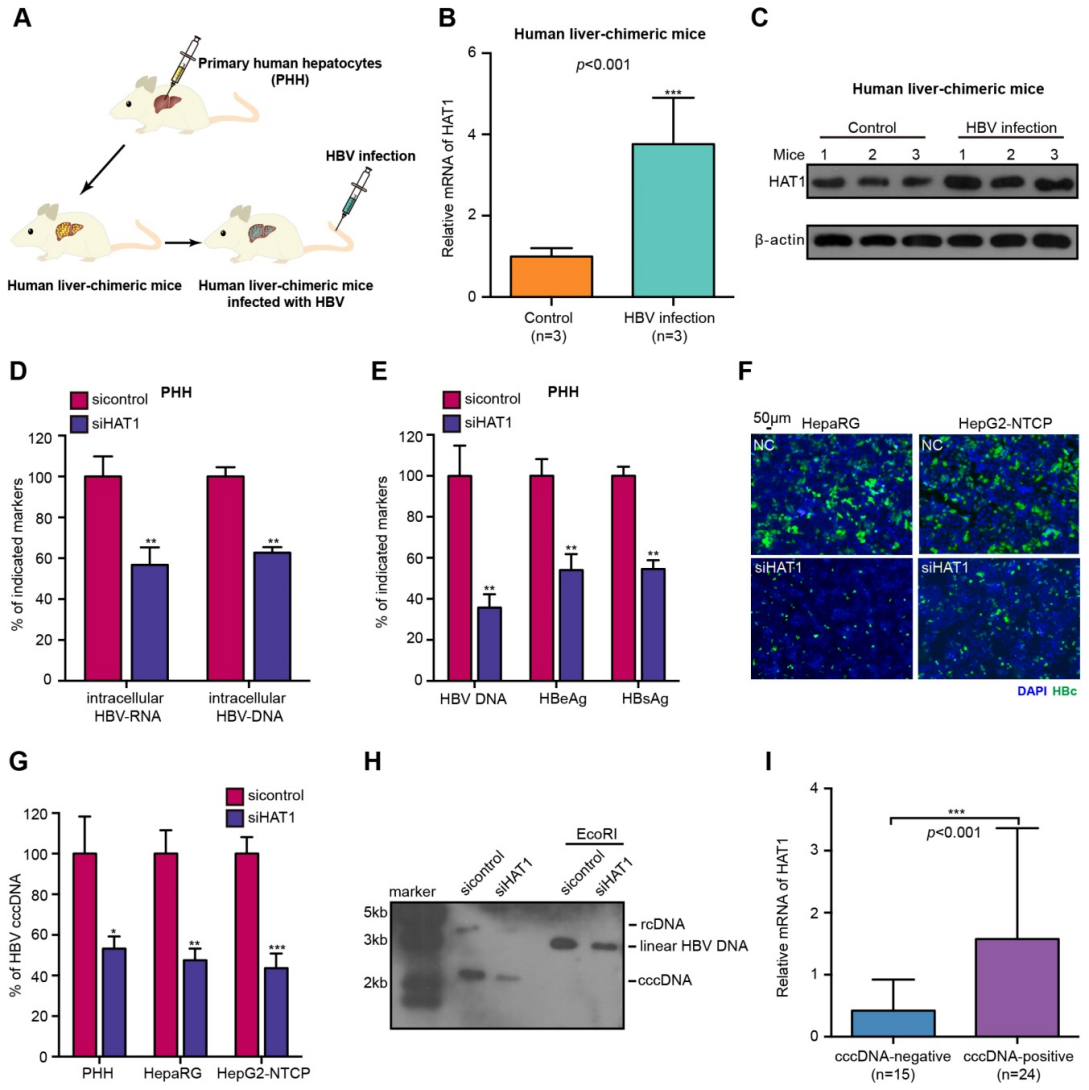
HAT1 contributes to HBV replication and cccDNA accumulation. (A) A model of the establishment of HBV-infected human liver-chimeric mice. (B and C) The mRNA and protein levels of HAT1 were examined by RT-qPCR and Western blot analysis in the liver of human liver-chimeric mice (n=3) and HBV-infected human liver-chimeric mice (n=3), respectively. (D-G) The PHH, dHepaRG and HepG2-NTCP cells were infected at MOI of 600 vp/cell with HBV and were continuously transfected with siRNA of HAT1 at -4, 0, and 4 dpi (days post-infection). (D) The intracellular HBV-RNA and intracellular HBV-DNA were detected 8 dpi by qPCR in the cells. (E) The levels of HBV DNA, HBeAg and HBsAg in the medium were measured by qPCR and ELISA in the cells. (F) The levels of HBcAg were assessed by immunofluorescence assays in the cells. (G) HBV cccDNA was analyzed 8 dpi by selective qPCR in the cells. (H) The dHepaRG cells were infected with HBV at MOI of 600 vp/cell and were continuously transfected with siHAT1 (0, 50 or 100 nM) at -4, 0, 4 and 8 dpi. HBV cccDNA was analyzed 4, 8 and 12 dpi by Southern blot analysis in the cells. (I) Relative mRNA levels of HAT1 were detected by RT-qPCR in cccDNA-positive non-tumorous liver tissues (n=24) and cccDNA-negative non-tumorous liver tissues (n=15). Mean ± SD of at least three experiments are shown, in which each experiment was designed by three replicates. Statistical significant differences are indicated: *P<0.05; **P<0.01; ***P<0.001.

The HBV cccDNA minichromosome organized by histones and non-histones is critical for HBV replication 10. In host cells, HAT1 is the sole known type B histone acetyltransferase, displaying a crucial role in the deposition of histone H3/H4 onto newly replicated DNA to form nucleosome 21, 28. Accordingly, we were interested in whether HAT1 contributed to the HBV replication. Firstly, we concerned the situation of HAT1 expression in the normal liver by using human liver-chimeric mouse model. The levels of albumin and HBV DNA in the mice were shown (Table S1). Strikingly, our data revealed that the mRNA and protein levels of HAT1 were significantly elevated in the liver of HBV-infected human liver-chimeric mice relative to that of control human liver-chimeric mice (n=3, *p*<0.001) (Figure 1A-C, Figure S1A). Then, we showed that the levels of HAT1 were also significantly increased in HBV transgenetic mice, HBV-infected cells and HBV-positive cells (Figure S1B-C). The siRNA and overexpression efficiency of HAT1 were verified in the cell lines used in this study (Figure S1D). The viability assays showed that siHAT1 did not affect the cell viability (Figure S1E).

Next, we further determined whether HAT1 affected the HBV replication. We adopted three HBV-infected cell lines and observed that HBV cccDNA and HBV DNA could be quantified and specifically detected (Figure S2A-B). As expected, the depletion of HAT1 reduced the levels of intracellular HBV-DNA and intracellular HBV-RNA in HBV-infected PHH, dHepaRG and HepG2-NTCP cells (Figure 1D, Figure S2C-D). Meanwhile, the levels of HBV DNA, HBeAg and HBsAg in the supernatant were decreased by siHAT1 (Figure 1E, Figure S2E-F). Immunostaining revealed that the levels of HBcAg were attenuated by siHAT1 in the cells (Figure 1F, Figure S2G). Impressively, qPCR and Southern blot analysis showed that the depletion of HAT1 strongly reduced the accumulation of cccDNA in HBV-infected PHH, dHepaRG and HepG2-NTCP cell lines (Figure 1G-H, Figure S2H), suggesting that HAT1 is crucial for HBV cccDNA accumulation. We further evaluated the relationships between HAT1 and HBV cccDNA in clinical liver tissues. Our data found that 39 samples were positive for HBV DNA in 43 non-tumorous liver tissues, in which 24 samples were positive for cccDNA. Interestingly, the expression levels of HAT1 were remarkably elevated in cccDNA-positive tissues (Figure 1I), supporting that HAT1 is able to modulate cccDNA in liver. Together, we conclude that HAT1 contributes to HBV replication and cccDNA accumulation.

### HAT1/CAF-1 signaling confers to the assembly of HBV cccDNA minichromosome

**Figure 2:**
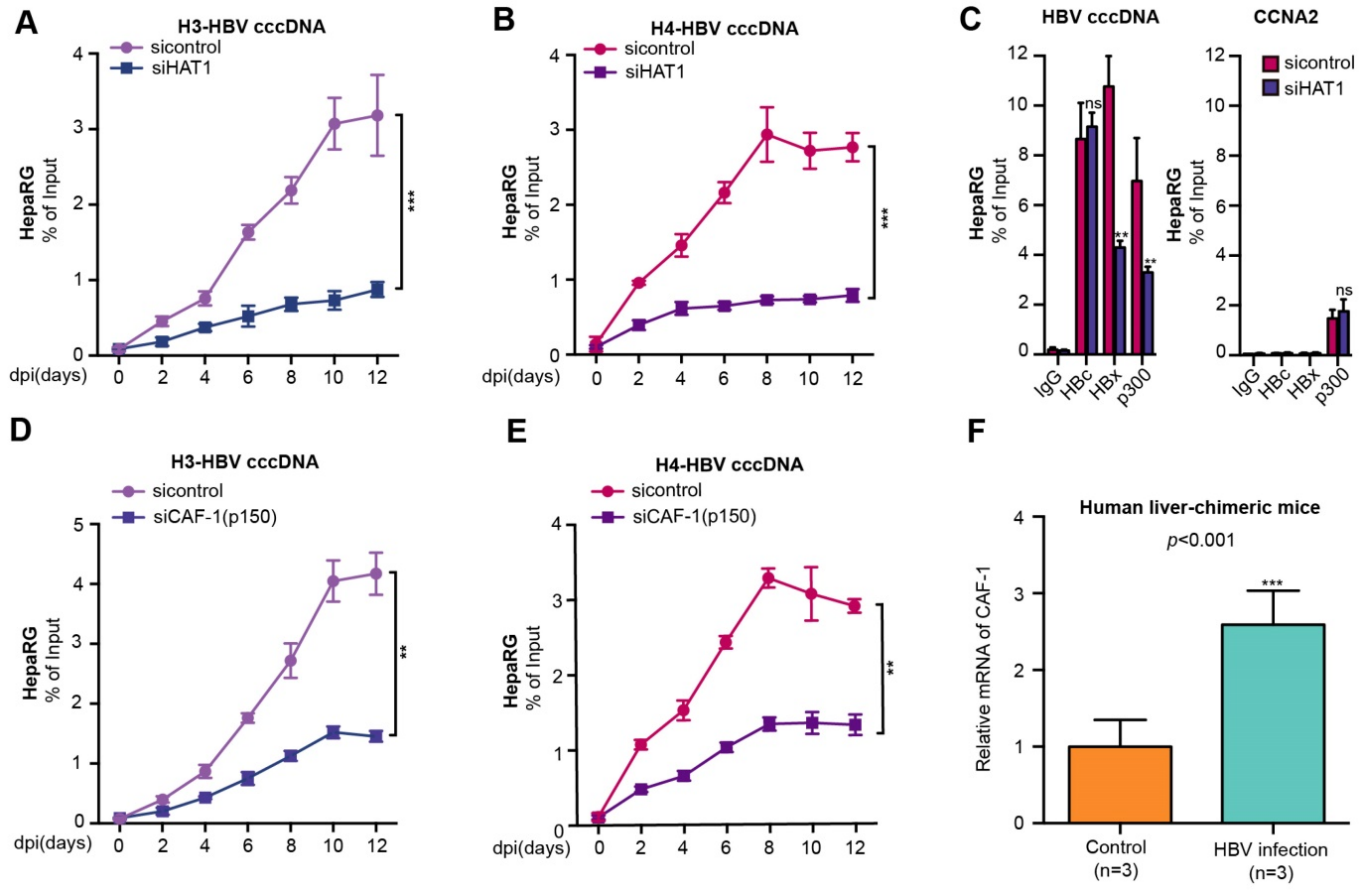
HAT1/CAF-1 signaling confers to the assembly of HBV cccDNA minichromosome. (A and B) The assembly of histone H3/H4 onto cccDNA was examined by ChIP-qPCR at indicated dpi in HBV-infected dHepaRG cells transfected with siHAT1. (C) The deposition of HBc, HBx and p300 onto cccDNA minichromosome or the promoter of CCNA2 was verified 8 dpi by ChIP-qPCR in HBV-infected dHepaRG cells continuously transfected with siHAT1. (D and E) The assembly of histone H3/H4 onto cccDNA was determined by ChIP-qPCR at indicated dpi in HBV-infected dHepaRG cells continuously transfected with siCAF-1 (p150) (100 nM) at -4, 0, 4 and 8 dpi. (F) The mRNA of CAF-1 were examined by RT-qPCR in the liver of human liver-chimeric mice (n=3) and HBV-infected human liver-chimeric mice (n=3). Mean ± SD of at least three experiments are shown, in which each experiment was designed by three replicates. Statistical significant differences are indicated: **P<0.01; ***P<0.001; ns, no significance.

Given that HAT1 signaling played critical role in the nucleosome assembly in host cells 21, we hypothesized whether HAT1 signaling was involved in the assembly of HBV cccDNA minichromosome (Figure S3A). Surprisingly, HAT1 knockdown notably reduced the assembly of histone H3/H4 onto cccDNA to form minichromosome, which was detected at 0, 2, 4, 6, 8, 10, 12 dpi (days after infection) in *de novo* HBV-infected dHepaRG and HepG2-NTCP cells (Figure 2A-B, Figure S3B-C), suggesting that HAT1 is critical for the assembly of histone H3/H4 onto cccDNA in the assembly of cccDNA minichromosome. The flow chart was shown (Figure S3D, left panel). The efficiency of siHAT1 was verified in the cells (Figure S3D, right panel).

Considering that HAT1 is able to modulate multiple acetylation sites including histone H3K27, H4K5 and H4K12, and plays crucial roles in mediation of host chromatin assembly by histone acetylation 21, 22, we further explored whether HAT1 contributed to the cccDNA minichromosome assembly by modification of H4K5 and H4K12. Interestingly, our data showed that overexpression of histone H4 increased the assembly of histone H4 onto cccDNA, whereas overexpression of K5 mutant (lysine K to arginine R) histone H4, K12 mutant (K to R) histone H4, and K5/K12 mutant (K to R) histone H4 failed to increase the assembly of histone H4 onto cccDNA in HBV-infected HepG2-NTCP cells, but the overexpression of K27 mutant histone H3 did not work in the system (Figure S3E-F).

In addition to histones, HBc is a structural component of HBV cccDNA minichromosome 10, 11. Some functional proteins, such as HBx and p300 also bind to HBV cccDNA minichromosome 18. Interestingly, our data showed that HAT1 knockdown decreased the deposition of HBx and p300, but not HBc, onto cccDNA minichromosome in the HBV-infected dHepaRG and HepG2-NTCP cells (Figure 2C, Figure S3G). As a negative control, the levels of HBc, HBx and p300 associated with the promoter of cyclin A2 (CCNA2) that was not regulated by HAT1, were not changed in the cells (Figure 2C, Figure S3G).

In the host cells, the acetylation pattern produced by HAT1 of newly synthesized histone H3/H4 is recognized by histone chaperones, such as CAF-1 and Asf1, to deposit the histones onto replicated DNA and form nucleosome 21, 24, 25. Surprisingly, we observed that the depletion of CAF-1, especially the p150 subunit, but not Asf1, impaired the assembly of histone H3/H4 onto cccDNA in the HBV-infected cells (Figure 2D-E, Figure S4A-D). The siRNA efficiency of CAF-1 (p150), CAF-1 (p60) and Asf1 were verified in the cell lines (Figure S4E-F). The viability assays showed that siCAF-1 (p150), siCAF-1 (p60) and siAsf1 did not affect the cell viability (Figure S4G). Interestingly, our data revealed that the mRNA and protein levels of CAF-1 were significantly elevated in the liver of HBV-infected human liver-chimeric mice relative to that of control human liver-chimeric mice (n=3, *p*<0.001) (Figure 2F, Figure S4H). Interestingly, siCAF-1 (p150) also reduced the deposition of HBx and p300, but not HBc, onto cccDNA minichromosome in the cells (Figure S4I-J). In addition, CAF-1 contributes to the accumulation of HBV cccDNA in the cells (Figure S4K). Taken together, we conclude that HAT1/CAF-1 signaling confers to the assembly of HBV cccDNA minichromosome by acetylating histone H4K5 and H4K12.

### HAT1 promotes histone acetylation on HBV cccDNA minichromosome

**Figure 3:**
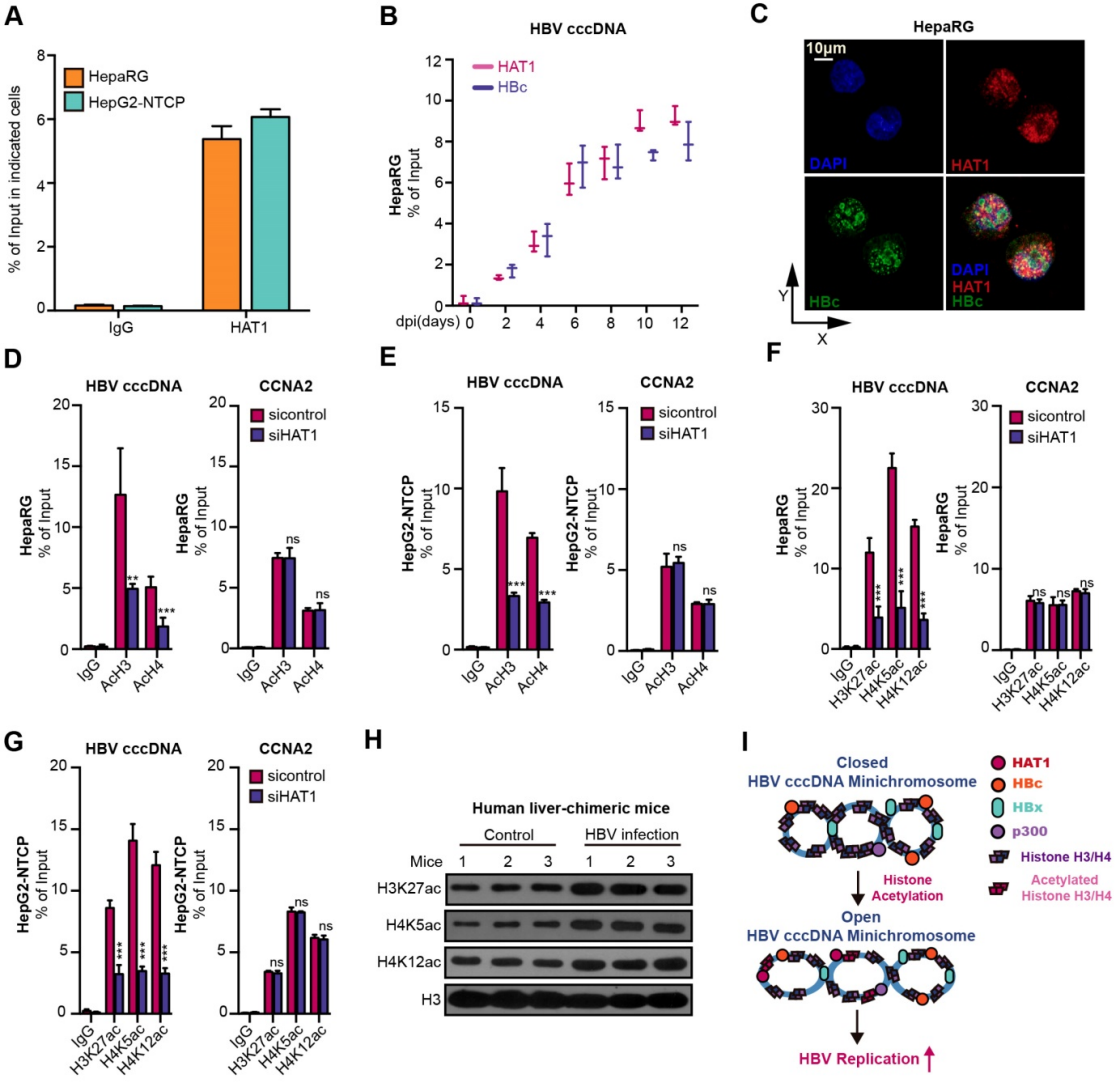
HAT1 promotes histone acetylation on HBV cccDNA minichromosome. (A) The deposition of HAT1 on cccDNA was measured by ChIP-qPCR in the HBV-infected dHepaRG and HepG2-NTCP cells. (B) The deposition of HAT1 and HBc on cccDNA was analyzed by ChIP-qPCR at indicated time in the HBV-infected dHepaRG cells. (C) The colocalization of HAT1 and HBc was assessed 8 dpi by confocal microscopy in the dHepaRG cells. (D-G) The acetylation of histone, including AcH3, AcH4, H3K27ac, H4K5ac and H4K12ac, associated to cccDNA minichromosome or the promoter of CCNA2 was verified 8 dpi by ChIP-qPCR in the dHepaRG and HepG2-NTCP cells. (H) The acetylation of histone H3K27, H4K5 and H4K12 were examined by Western blot analysis in the liver of human liver-chimeric mice (n=3) and HBV-infected human liver-chimeric mice (n=3). (I) A model for the function of HAT1 in promoting histone acetylation of cccDNA minichromosome was shown. Mean ± SD of at least three experiments are shown, in which each experiment was designed by three replicates. Statistical significant differences are indicated: **P<0.01; ***P<0.001; ns, no significance.

Globally, the acetylation of histones associated with HBV cccDNA parallels the active HBV replication 29, 30. Interestingly, we uncovered that HAT1 anchored onto the cccDNA minichromosome in the cells (Figure 3A-B). Moreover, confocal microscopy displayed a co-localization of HAT1 and HBc in the nucleus of HBV-infected dHepaRG and HepG2-NTCP cells (Figure 3C, Figure S5A). Significantly, our data showed that the depletion of HAT1 reduced the acetylation of histone H3/H4, specifically at H3K27, H4K5 and H4K12 on cccDNA minichromosome in the HBV-infected dHepaRG and HepG2-NTCP cells (Figure 3D-G), suggesting that HAT1 is able to modulate the acetylation of histone H3/H4 onto cccDNA minichromosome. It has been reported that the binding of RNA Polymerase II (Pol2) on to cccDNA minichromosome can reveal the levels of transcription of cccDNA minichromosome 30. As expected, siHAT1 could reduce the binding of Pol2 to cccDNA minichromosome in the cells (Figure S5B-C). Importantly, our data showed that overexpression of histone H3 and H4 increased the HBV replication in HBV-infected dHepaRG and HepG2-NTCP cells, whereas overexpression of K5 mutant (lysine K to arginine R) histone H4, K12 mutant (K to R) histone H4, and K27 mutant (K to R) histone H3 failed to increase the HBV replication in the cells (Figure S5D-I). Meanwhile, the acetylation of histone H3K27, H4K5 and H4K12 was elevated in HBV-infected human liver-chimeric mice, suggesting that the acetylation of histone H3K27, H4K5 and H4K12 is positively relative to HBV (Figure 3H, Figure S5J). Taken together, we conclude that HAT1 contributes to the histone acetylation on HBV cccDNA minichromosome for an active status, which is summarized in a mini-model (Figure 3I).

### HAT1 is recruited to cccDNA minichromosome by lncRNA HULC-scaffold HBc

**Figure 4:**
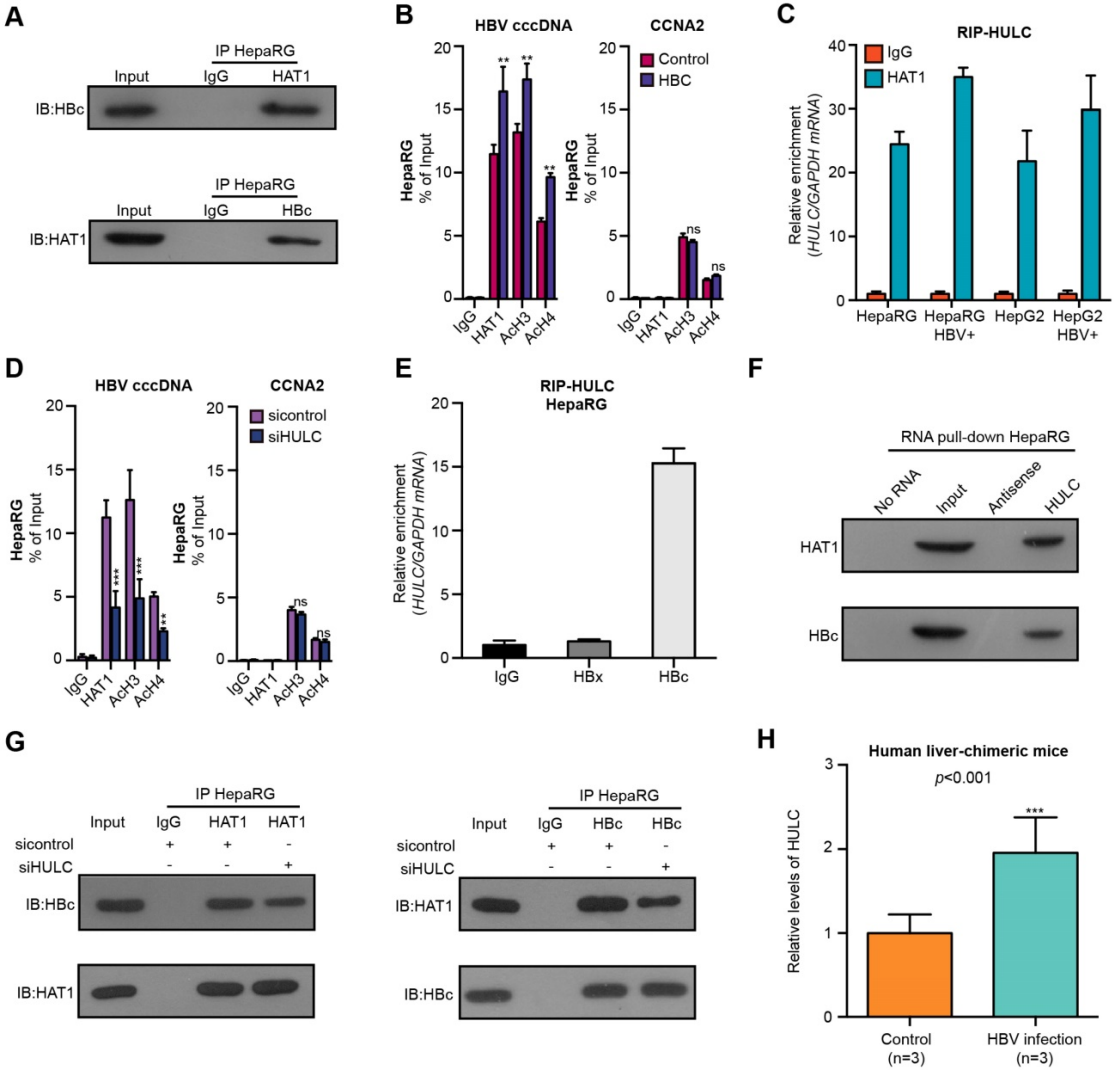
HAT1 is recruited to cccDNA minichromosome by lncRNA HULC-scaffold HBc. (A) The combination of HAT1 and HBc was measured 8 dpi by immunoprecipitation assays in the HBV-infected dHepaRG cells. (B) The deposition of HAT1 and acetylation of histone including AcH3 and AcH4 on cccDNA minichromosome or the promoter of CCNA2 were verified 8 dpi by ChIP-qPCR in HBV-infected dHepaRG cells continuously transfected with pcDNA 3.1-HBC (2 μg/well) at -4, 0, and 4 dpi. (C) The interaction of HULC with HAT1 was examined by RIP-qPCR assays in the indicated cells. (D) The deposition of HAT1 and acetylation of histone including AcH3 and AcH4 on cccDNA minichromosome or the promoter of CCNA2 were analyzed 8 dpi by ChIP-qPCR in HBV-infected dHepaRG cells continuously transfected with siHULC (100 nM) at -4, 0, and 4 dpi. (E) The combination of HULC with HBc or HBx was examined by RIP-qPCR assays 8 dpi in HBV-infected dHepaRG cells. (F) The interaction of HULC with HBc or HAT1 was assessed 8 dpi by RNA pull-down assays followed by Western blot analysis in HBV-infected dHepaRG cells. (G) The combination of HAT1 and HBc was analyzed 8 dpi by immunoprecipitation assays in HBV-infected dHepaRG cells transfected with siHULC. (H) The levels of lncRNA HULC were examined in the liver of human liver-chimeric mice (n=3) and HBV-infected human liver-chimeric mice (n=3) by RT-qPCR. Mean ± SD of at least three experiments are shown, in which each experiment was designed by three replicates. Statistical significant differences are indicated: **P<0.01; ***P<0.001; ns, no significance.

Next, we further identified the mechanism by which HAT1 was recruited and bound to cccDNA minichromosome. Interestingly, we observed the interaction of HAT1 with HBc by immunoprecipitation assays and GST pull-down assays (Figure 4A, Figure S6A). Moreover, the overexpression of HBC increased the recruitment of HAT1 and the histone acetylation on cccDNA minichromosome in the HBV-infected dHepaRG and HepG2-NTCP cells (Figure 4B, Figure S6B-C). LncRNAs have been primarily implicated in transcriptional regulation by serving as scaffolds for assembling transcriptional regulators 26. Accordingly, we raised a bold hypothesis: the lncRNA as a scaffold may consolidate the interaction of HAT1 with HBc for the deposition of HAT1 onto cccDNA. It has been reported that lncRNAs including HULC, HOTAIR and LncTCF7 are highly expressed in liver cancer samples 27, 31-33. Surprisingly, RIP analysis identified that the lncRNA HULC, but not HOTAIR and LncTCF7, could bind to HAT1 in the cells (Figure 4C, Figure S6D-E). Moreover, the depletion of HULC reduced the deposition of HAT1 and the histone acetylation on cccDNA minichromosome (Figure 4D, Figure S6F-G). RIP analysis further confirmed the interaction of HULC with HBc in the cells (Figure 4E, Figure S6H-I). RNA pull-down assays followed by Western blot analysis verified the combination of HULC with HAT1 or HBc in the cells (Figure 4F). Importantly, immunoprecipitation assays showed that the HULC knockdown attenuated the interaction of HAT1 with HBc in the system (Figure 4G, Figure S6J). Functionally, HULC knockdown could block the increase of intracellular HBV-DNA and HBeAg mediated by HAT1 in HBV-infected cells (Figure S6K-L). Interestingly, we found that the levels of lncRNA HULC were significantly elevated in the liver of HBV-infected human liver-chimeric mice relative to that of control human liver-chimeric mice (n=3, *p*<0.001) (Figure 4H). RIP analysis further confirmed the interaction of HULC with HAT1 and HBc in the liver of HBV-infected human liver-chimeric mice (n=3) (Figure S6M). We conclude that HAT1 anchors to the cccDNA minichromosome through interaction with HBc, in which HULC serves as a scaffold in the complex of HAT1/HULC/HBc, to modulate acetylation of histones on cccDNA minichromosome (Figure S6N).

### HBV stimulates HAT1 promoter through HBx-co-activated transcriptional factor Sp1

**Figure 5:**
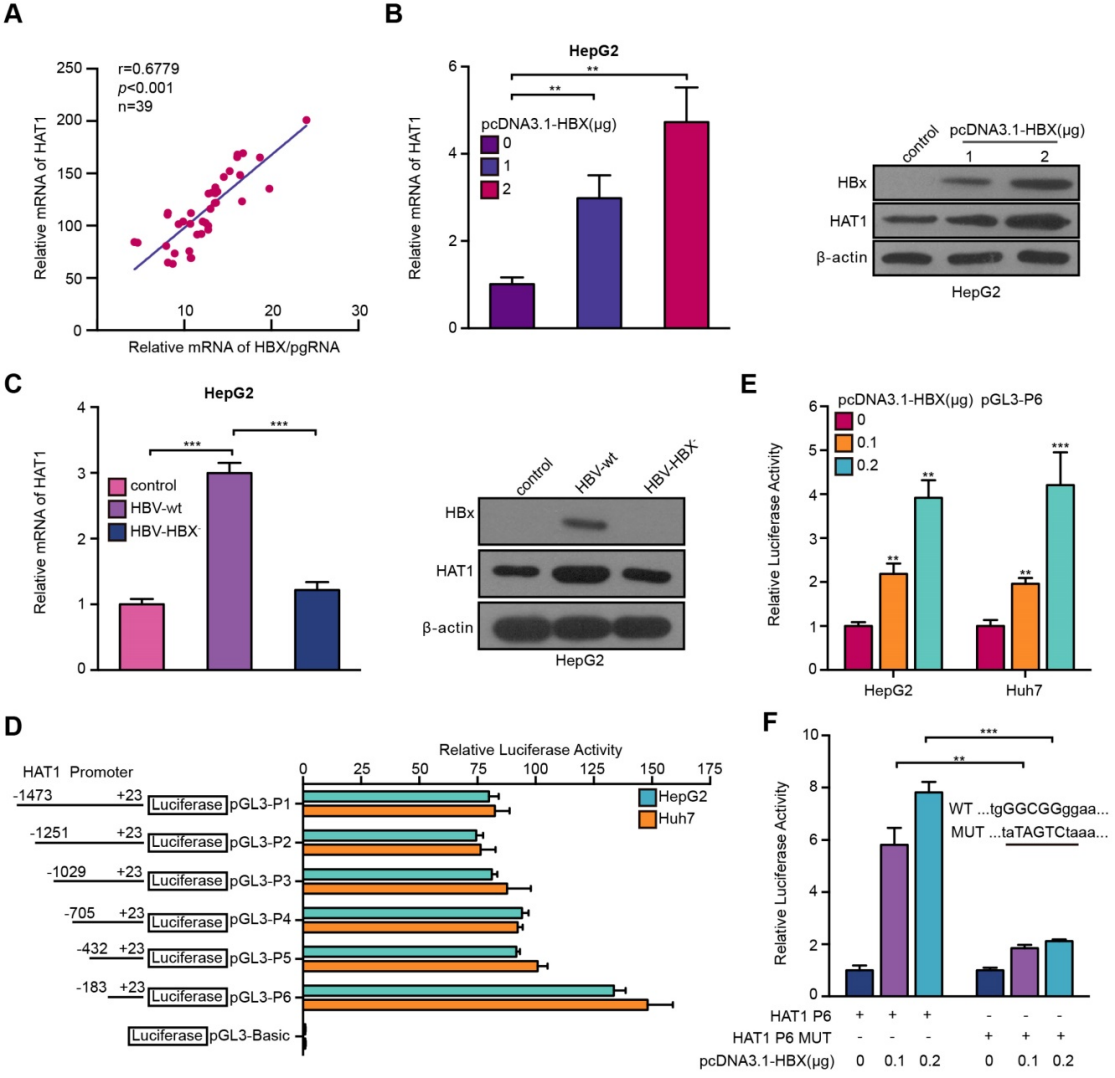
HBV stimulates HAT1 promoter through HBx-co-activated transcriptional factor Sp1. (A) Correlation between HAT1 and HBX/pgRNA was examined by RT-qPCR in HBV-positive clinical HCC tissues. (B) The expression levels of HAT1 were assessed by RT-qPCR and Western blot analysis in HepG2 cells transfected with pcDNA3.1-HBX (0, 1 or 2 μg/well). (C) The expression levels of HAT1 were tested by RT-qPCR and Western blot analysis in HepG2 cells transfected with wild-type pCH-9/3091 (HBV-wt) or HBX deficient pCH-9/3091 (HBV-HBX^-^). (D) Luciferase activities of HAT1 promoter were analyzed in the cells. HepG2 and Huh7 cells were transfected with pGL3-Basic or reporter constructs containing various lengths of the 5'-flanking region of the HAT1 gene, as indicated (pGL3-P1, pGL3-P2, pGL3-P3, pGL3-P4, pGL3-P5 and pGL3-P6, respectively). (E) Luciferase activities of HAT1 P6 promoter were analyzed in HepG2 and Huh7 cells transfected with control vector or pcDNA3.1-HBX. (F) Luciferase activities of HAT1 P6 wild type or HAT1 P6 Sp1 binding site mutant type were determined in HepG2 cells transfected with pcDNA3.1-HBX. The illustration of the responsive region to Sp1 in HAT1 promoter was shown. Mean ± SD of at least three experiments are shown, in which each experiment was designed by three replicates. Statistical significant differences are indicated: **P<0.01; ***P<0.001.

Given that the interaction of virus-host was crucial for HBV life cycle in liver 14, 29, we asked whether the HBV reversely affected HAT1 in the cells. It has been reported that HBx can regulate multiple genes as a co-activator of transcription factor. Therefore, we supposed that HBV might modulate HAT1 through HBx in a positive feedback manner. Clinically, the expression levels of HAT1 positively correlated with those of HBX/pgRNA in the HBV positive HCC tissues (Figure 5A). RT-qPCR and Western blot analysis showed that HBx dose-dependently up-regulated HAT1 in HepG2 cells (Figure 5B, Figure S7A). In addition, the levels of HAT1 were reduced in HepG2 cells transfected with HBX deficient HBV (HBV-HBX^-^) (Figure 5C, Figure S7B), supporting that HBx is able to up-regulate HAT1 in the liver cells.

It has been reported that HBx serves as a trans-activator modulated genes transcription 34. Accordingly, we first probed whether HBx increased HAT1 by activating its promoter. To this end, we first identified the HAT1 promoter core region. Luciferase reporter gene assays indicated that the region of -183/+23 (pGL3-P6) exhibited the maximum luciferase activities in the cells (Figure 5D), indicating that pGL3-P6 is the core region of HAT1 promoter. Functionally, overexpression of HBX was able to increase the luciferase activities of pGL3-P6 in the HepG2 and Huh7 cells (Figure 5E). Next, bioinformatics analysis demonstrated that the promoter region 183/+23 contained Sp1 element. It has been reported that HBx is able to interact with Sp1 to regulate transcription 35. As expected, our results showed that HBx could stimulate HAT1 promoter by co-activating Sp1 (Figure 5F, Figure S7C-I). Overall, we conclude that HBV induces HAT1 through HBx-co-activated transcriptional factor Sp1.

## Discussion

Chronic infection of HBV greatly increases the risk for terminal liver disease 29. Current therapies rarely achieve a cure due to the refractory nature of HBV cccDNA minichromosome 10. The host molecules that modulate the cccDNA minichromosome are potential targets of anti-HBV therapy. However, the modification of HBV cccDNA minichromosome, especially the assembly, accumulation and epigenetic regulation, remains unclear. In this study, we tried to identify the host molecules that modulated HBV cccDNA minichromosome in liver.

Firstly, we concerned the situation of HAT1 expression in the normal liver by using human liver-chimeric mouse model. Interestingly, we observed that the levels of HAT1 were significantly increased in the liver of HBV-infected human liver-chimeric mice. It suggests that the expression of HAT1 is positively related to the HBV replication. Importantly, we validated that HAT1 could promote the HBV replication and cccDNA accumulation. It has been reported that HAT1 mediated host chromatin assembly by acetylating histone H4 at the site of H4K5 and H4K12 21. Interestingly, we firstly uncovered that HAT1 modulated HBV cccDNA minichromosome assembly by acetylation of histone H4K5 and H4K12. The acetylation pattern produced by HAT1 of newly synthesized histone H3/H4 is recognized by histone chaperones, such as CAF-1 and Asf1, to deposit the histones onto replicated DNA and form nucleosome in the host cells 21. Accordingly, we identified that histone chaperone CAF-1 was also involved in the assembly and accumulation of cccDNA minichromosome.

In recent years, a number of epigenetic markers on the HBV cccDNA minichromosome associated with viral transcription have been identified 14, 15, 30. And the role of methylation of cccDNA-bound histones in the regulation of HBV transcription has been well recognized 17. However, the role of histone acetylation in modulation of HBV cccDNA minichromosome remains elusive. Interestingly, our findings suggest that HAT1 is a new modulator for the site-specific histone acetylation of HBV cccDNA minichromosome. We uncovered that the complex of HBc/HAT1/HULC played critical roles in the epigenetic modification of HBV cccDNA minichromosome. Next, we concerned whether HBV could affect HAT1 during the process of interaction of host-virus in liver. As expected, we observed that HBV was able to up-regulate HAT1 by HBx-controlled transcriptional factor Sp1, providing new evidence that HBV hijacks the host key factors to enhance itself in a positive feedback mechanism.

**Figure 6:**
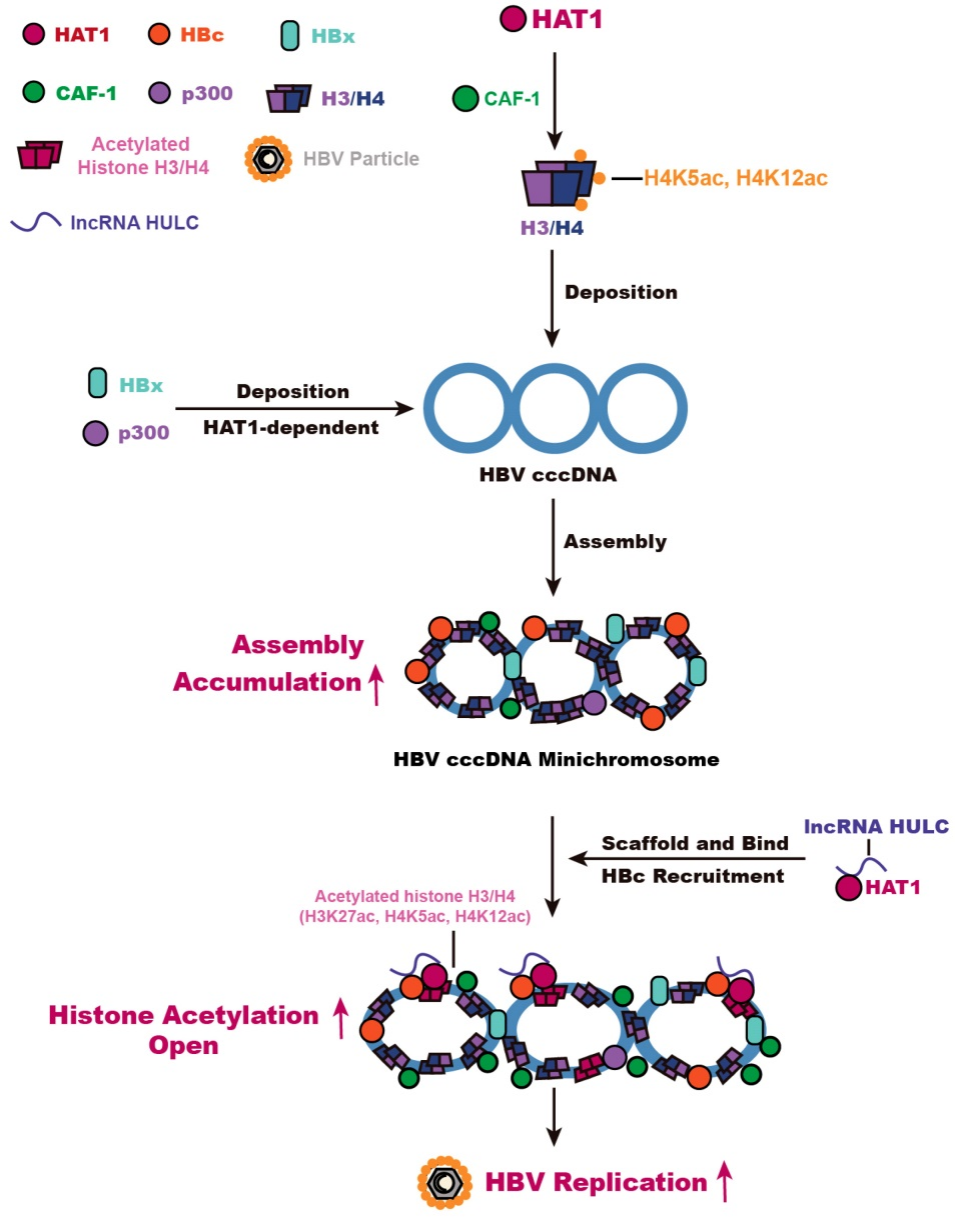
HAT1 signaling confers to the regulation of HBV cccDNA minichromosome. In this model, HAT1/CAF-1 signaling, which is the host nucleosome assembly machinery, contributes to the assembly of cccDNA minichromosome by acetylating histone H4 at the sites of K5 and K12, leading to the accumulation of cccDNA. HAT1 is recruited to the cccDNA minichromosome through interacting with HBc, in which lncRNA HULC serves as a scaffold in the complex of HAT1/HULC/HBc for acetylation of histones on cccDNA minichromosome to activate HBV transcription.

Overall, we summarize a model for HBV cccDNA minichromosome mediated by HAT1 signaling (Figure 6). A host nucleosome assembly HAT1 signaling contributes to assembly and epigenetic regulation of HBV cccDNA minichromosome, leading to the HBV replication. Our finding provides new insights into the mechanism by which the host HAT1 signaling modulates episomal DNA viruses.

## Materials and Methods

### HBV Inocula, Cell Cultures and HBV Infection

HBV inocula were prepared as described 8, 13. Shortly, media from HepAD38 cells at day's 7-15 post-induction of HBV by depletion of tetracycline were recovered every 3 days. Media were cleared through a 0.45 μm filter and precipitated with 10% PEG 8000 and 2.3% NaCl. The precipitates were washed and resuspended with medium at 200-fold concentration. HBV DNA was quantified by real-time PCR.

Primary human hepatocytes (PHHs) were purchased from RIDL (Shanghai, China), cultured and infected with HBV as described 36, 37. Briefly, PHH was maintained in William's E medium (Gibco-Invitrogen) with 5% FCS, 7× 10^-5^ M hydrocortisone hemisuccinate, 5 μg/ml insulin and 2% DMSO (Sigma-Cell Culture reagent). HepaRG differentiation (dHepaRG) was been described previously 38. Briefly, cells were maintained for 2 weeks in standard medium then for at least 2 weeks in standard medium with 1.8 % DMSO and EGF (5 ng/ml) (PeproTech-Tebu, France). An expression plasmid for hNTCP was transfected into HepG2 cells with TransIT-LT1 (Mirus, USA) according to the manufacturer's instructions to establish HepG2-NTCP cells 1, 39. HBV infections were also performed as described 8, 12, 15. Briefly, HBV infection was carried out with HBV purified from supernatant of HepAD38 cells by heparin affinity chromatography and subsequent concentration via sucrose gradient ultracentrifugation (at a MOI of 600 vp/cell) in PHH, dHepaRG and HepG2-NTCP cells. Infection was performed by using 5% PEG 8000 and William's E medium contain 10% FBS, Penicillin/streptomycin, Human insulin (350 μl, sigma I9278, USA), Hydrocortison (5 μg/ml, sigma H2270), and 1.8% DMSO (sigma, 2650).

HepG2, HepG2-X and HepG2.2.15 cell lines were maintained in Dulbecco's modified Eagle' s medium (Gibco, Grand Island, NY, USA). Huh7 cells were cultured in RPMI Medium 1640 (Gibco, Grand Island, NY, USA). HepAD38 cell line regulated HBV replication through the presence or absence of tetracycline in the culture medium, which were cultured in DMEM/F12 medium (Life Technologies, Carlsbad, CA) supplemented with 10% heat-inactivated FCS, 100 U/ml penicillin, 100 μg/ml streptomycin, 100 μg/ml kanamycin, 400 μg/ml G418, and with 0.3 μg/ml tetracycline (for inhibition of HBV replication) or without any tetracycline (for induction of HBV replication). The immortalized human normal liver LO2 cells were cultured in RPMI 1640 medium (Life Technologies, Carlsbad, CA). HepG2 cells were transfected with pCH-9/3091 (HBV 1.1× plasmids expressing the whole HBV genome) to induce the HBV life cycle. The transfection was performed using Lipofectamine RNAiMAX (Thermo Fisher Scientific, USA) and Lipofectamine MessengerMAX (Thermo Fisher Scientific, USA) according to the manufacturer's protocol in PHH, dHepaRG and HepG2-NTCP cell lines. The cell viability was measured using the MTS assay (Promega, USA).

### Generation of Human Liver-chimeric Mice

The human liver-chimeric mice were generated by VITALSTAR (Beijing, China). Primary human hepatocytes (PHHs) were transplanted into 3-week-old urokinase-type plasminogen activator/severe combined immunodeficient beige (uPA/SCID-bg) mice (male and female) by intrasplenic injection as described 8, 40. Engraftment and viability of PHHs were assessed by quantification of human serum albumin by enzyme-linked immunosorbent assay (Human Albumin ELISA kit, Immunology Consultants Lab, Portland, USA). Then, the uPA/SCID-bg mice were infected with 2.5E+08 IU/ml (0.2ml/mouse) HBV particles from the supernatant of HepAD38 cells (tet-off) and sacrificed 8 weeks after virus inoculation. Serum HBV load in the mice was determined by quantitative PCR (Da An Gene, Guangzhou, China) before sacrifice. The information of human liver-chimeric mice was shown in Table S1.

### Patient samples

Forty-three non-tumorous liver tissues from HCC patients utilized in this study were immediately obtained from Tianjin First Center Hospital (Tianjin, P.R. China) and Tianjin Medical University Cancer Institute and Hospital (Tianjin, P.R. China) after surgical resection. Clinicopathological information about the patients was obtained from patient records, and was summarized in Table S2. Written consents approving the use of their tissues for research purposes after operation were obtained from patients. The Institute Research Ethics Committee at the Nankai University approved the study protocol.

### RNA extraction, reverse-transcription polymerase chain reaction (RT-PCR), and quantitative real-time PCR (RT-qPCR)

Total RNA was extracted from cells (or liver tissues from HBV-Tg mice and patient) using Trizol reagent (Invitrogen, Carlsbad, CA). First-strand cDNA was synthesized as reported before. RT-qPCR was performed by a Bio-Rad sequence detection system according to the manufacturer's instructions using double-stranded DNA-specific SYBR GreenPremix Ex TaqTM II Kit (TaKaRa, Ohtsu, Japan). Experiments were conducted in duplicate in three independent assays. Relative transcriptional folds were calculated as 2^-∆∆Ct^. GAPDH was used as an internal control for normalization. The expression levels of HAT1, CAF-1 and HULC were analyzed by RT-qPCR in liver tissues of human liver-chimeric mice and HBV infected human liver-chimeric mice. All the primers used are listed in Table S3.

### HBV transgenetic mice and tissue analysis

HBV transgenic mice BALB/c (HBV-Tg) containing HBV genome S, pre-S, and X domains were purchased from VITALRIVER experiment animal company (Beijing, China), BALB/c mice were also purchased from VITALRIVER experiment animal company. Animals were maintained under specific pathogen free condition. Mice were sacrificed and serum and liver tissue were obtained 13, 34, 41. The Institute Research Ethics Committee at the Nankai University approved the study protocol.

### HBV cccDNA isolation

Selective extraction of HBV cccDNA from the cells was achieved by a modified method as previously described 12, 15, 17. The cells were lysed in lysis buffer A (50 mM Tris-HCl pH 7.4, 1 mM EDTA, 1% NP-40) containing complete protease inhibitor cocktail for 30 min on ice. After centrifugation, the pelleted nuclei were resuspended in lysis buffer B (10 mM Tris-HCl, 10 mM EDTA, 150 mM NaCl, 0.5% SDS, Proteinase K 0.5 mg/ml) and incubated overnight at 37 °C. Nucleic acids were purified by phenol-chloroform (1:1) extraction and ethanol precipitation. HBV cccDNA was further used for analysis using qPCR and Southern blot analysis.

### Southern blot analysis of HBV cccDNA

For detection of cccDNA by Southern blot, the extracted HBV cccDNA sample was subjected to 1.2% agarose gel electrophoresis and transferred onto Amersham Hybond-N+ membrane (GE Healthcare). The Hybond-N+ membrane was cross-linked in a UV cross linker chamber with UV energy dosage at 1500 mJ and followed by being probed with “DIG-labeled probes of linear HBx DNA fragments” for 24 h, 37 °C. And the membrane was blocked and incubated with Anti-Digoxigenin-AP (dilute Anti-Digoxigenin-AP 1:10 000 (75 mU/ml) in Blocking solution) for overnight at 4 °C. After washing for 15 min, we place membrane with DNA side facing up on a development folder (or hybridization bag) and apply 1 ml CSPD ready-to-use (bottle 5). Immediately cover the membrane with the second sheet of the folder to spread the substrate evenly and without air bubbles over the membrane. Incubate for 5 min at 15-25 °C. When indicated, isolated DNA was digested with EcoRI that linearizes the cccDNA.

### HBV cccDNA-ChIP

ChIP experiments were carried out in infected cells 8 days post-infection as described with minor modifications 15. Briefly, cells were fixed with 1% formaldehyde for 10 min at 37 °C and quenched with 0.125 M Glycine. For nuclear extracts preparation, cells were lysed in buffer A (0.25% Triton X-100, 10 mM Tris pH 8, 0.5 mM pefablock, EDTA-free protease inhibitors (Roche)). After centrifugation, nuclei were washed in buffer B (0.2 M NaCl, 10 mM Tris pH 8, 0.5 mM pefablock, EDTA-free protease inhibitors), centrifuged and lysed in nuclei lysis buffer (1% SDS, 10 mM EDTA, 50 mM Tris pH 8, 0.5 mM pefablock, EDTA-free protease inhibitors). After sonication, lysates were diluted 1:10 with 0.01% SDS, 1% Triton X-100, 1.2 mM EDTA, 16.7 mM Tris pH 8, 167 mM NaCl, 0.5 mM pefa-block and EDTA-free protease inhibitors. Chromatin was then subjected to overnight immunoprecipitation at 4 °C using 2-5 μg of antibodies listed in the Table S5. Negative controls with nonspecific immunoglobulin (Millipore PP64B) were included in each experiment. Immune complexes were incubated with protein A/protein G agarose beads at 4 °C, washed, and eluted in 1% SDS, 0.1% NaHCO3. Immunoprecipitated DNA was quantified by qPCR using cccDNA specific primers or specific primers for the cyclin A2 promoter (hCCNA2), a negative control. Samples were normalized to input DNA using the ∆Ct method were ∆Ct = Ct (input) - Ct (immunoprecipitation) and calculated as percentage of the input. Results were expressed as the average of at least three independent experiments. Standard error of the mean (SEM) was indicated. Statistical differences were analyzed by Student's *t* test.

### Analysis of HBV replication intermediates

Secretion of HBsAg into the supernatants of cultured cells was measured by Diagnostic Kit according to the manufacturer's instructions (Kehua Bio-engineering, Shanghai, China). The cut-off value (COV) for HBsAg analysis was indicated as: COV =OD (negative control)/0.100 17. HBeAg in the supernatants of cultured cells was measured by Diagnostic Kit according to the manufacturer's instructions (Kehua Bio-engineering, Shanghai, China). The cut-off value for HBeAg analysis was indicated as: COV= OD (negative control)*2.1(0<ODNC≤0.05, COV=0.05*2.1=0.105; 0.05<ODNC≤0.1; ODNC>0.1, invalidation) 17. The HBV DNA in the serum of mice or in the supernatants of cultured cells was extracted using the Blood & Cell Culture DNA kit (QIAGEN, Germany) following the manufacturer's instructions. The qPCR was used to quantify HBV DNA copies in the cells and clinical liver tissues according to a diagnostic kit for quantification of HBV DNA (Da An Gene, Guangzhou, China) by a Bio-Rad sequence detection system 13. Total DNA was purified from infected cells using a “Tissue kit” (Macherey Nagel, Düren, Germany). Total RNA was extracted from infected cells (NucleoSpin® RNA II kit, Macherey Nagel) and transcribed into cDNA. HBV-DNA and HBV-RNA were detected using specific PCR primers as described 12, 15.

### HBV cccDNA quantification

Before lysis, cell number was counted using Cell Counting Chamber Set (Qiujing, Shanghai, China). The procedure of the isolation of HBV cccDNA from the cells has been reported. Quantification of HBV cccDNA was performed by qPCR described with minor modifications 12, 15, 39. In brief, HBV-infected cells were lysed for 4 hr at 65°C in lysis buffer (50 mM Tris-HCl, pH 8.0, 50 mM EDTA, 100 mM NaCl, 1% SDS) supplemented with proteinase K (200 μg/ml) and followed by phenol-chloroform extraction. Aliquots of each DNA extracted from cell pellets were treated for 1 hour at 37°C with 10 U Plasmid-safe ATP-dependent DNase (Epicentre, Madison, WI, USA). The qPCR experiments were performed in a Mastercycler ep realplex (Eppendorf, Germany) using a 20 μl reaction volume containing 2 μl digested HBV DNA. Primers (forward, 5′-GCCTATTGATTGGAAAGTATGT-3′; reverse, 5′-AGCTGAGGCGGTATCTA-3′), which were used to amplify the cccDNA, were listed in Table S3.

### Immunofluorescence assays

To analyze the expression levels of HBcAg and the colocalization of HBc and HAT1, we used the HBc monoclonal antibody (Abcam ab8639, USA) and HAT1 monoclonal antibody (Abcam ab194296, USA), which were listed in Table S5. Virus infected cells in 48-well plates were washed three times with pre-cooled PBS and fixed by 4% paraformaldehyde for 10 min, followed by permeabilization for 10 min at room-temperature with 0.5% Triton X-100. After incubation for 1 h with 3% BSA for blockade of nonspecific binding, primary antibodies were added for incubation for 1 h at 37 °C. The bound antibodies were visualized by incubation with secondary antibodies (Alexa Fluor 488 donkey anti-mouse IgG or Alexa Fluor 546 anti-mouse IgG). Images were acquired using a Nikon A1-R confocal microscope or a Nikon Eclipse Ti Fluorescence Microscopy. The quantification of HBc fluorescence was analyzed by ImageJ software.

### Luciferase reporter gene assays

Luciferase reporter gene assays were performed using the Dual-Luciferase Reporter Assay System (Promega, Madison, WI) according to the manufacturer's instructions. Cells were transferred into 24-well plates at 3 × 10^4^ cells per well. After 24 h, the cells were transiently co-transfected with 0.1 μg/well of pRL-TK plasmid (Promega, Madison, WI, USA) containing the Renilla luciferase gene used for internal normalization, and various constructs containing different lengths of the HAT1 5'-flanking region or pGL3-Basic. The luciferase activities were measured as previously described 34. All experiments were performed at least three times. Bioinformatics analysis of HAT1 promoter was performed by PROMO (http://alggen.lsi.upc.es) and GPMiner (http://gpminer.mbc.nctu. edu.tw).

### GST pull-down assays

According to standard protocols 13, recombinant GST-HAT1 and His-HBc proteins were produced in Escherichia coli BL21 cells and purified with glutathione-Sepharose 4B (GE Healthcare, Waukesha, WI) or Ni-NTA resin (GE Healthcare, Waukesha, WI), respectively. Ten micrograms of GST or GST fusion proteins were incubated at 4 °C overnight with 10 μg purified His-HBc and 20 μl glutathione-Sepharose beads. Supernatants were collected as input and the Sepharose beads were then extensively washed 6 times with lysis buffer and resuspended in SDS loading buffer and boiled. Then, the sample buffer was loaded in 12% SDS-PAGE for detection with anti-His antibody.

### RNA immunoprecipitation assays

RIP assays were performed in native conditions as described 32. Briefly, cell nuclei were pelleted and lysed. The lysates were passed through a 27.5 gauge needle 4 times to promote nuclear lysis. The supernatant was incubated with 4 μg primary antibody with 40 μl protein G-conjugated agarose beads (Millipore). The RNA/antibody complex was washed by NT2 buffer (50 mmol/L Tris-HCl, pH 7.4, 150 mmol/L NaCl, 1 mmol/L MgCl2, 0.05% NP-40). The RNA was extracted by TRIzol (Invitrogen) according to the manufacturer's protocol and subjected to RT-qPCR analysis. The interaction of HULC with HAT1 and HBc was analyzed by RIP in the liver from HBV infected human liver-chimeric mice. Institute Research Ethics Committee at the Nankai University approved the study protocol.

### Western blot analysis

Total protein lysates were extracted from hepatoma cells with RIPA buffer. Protein concentrations were measured using the BCA protein Quantification Kit (YEASEN, China), and 20-50 μg protein extracts were subjected to SDS-PAGE. Then proteins were transferred to a nitrocellulose membrane, blocked with 5% non-fat milk and incubated with first antibodies for 1h at R.T. After incubation with secondary antibody against mouse (1:10,000) or rabbit (1: 10,000) for 1h at 37℃, the membrane was visualized by ECL Western Blotting Detection Kit (GE Healthcare, Waukesha, WI). Western blot analysis-Nucleocytoplasmic fractionation was performed as described previously 42-44. In brief, cells were collected and resuspended in 1: 5 diluted buffer A (50 mM Hepes, pH7.4; 1 mM EDTA, 10 mM mannitol; 1 mM DTT, 2 μg/ml aprotinin; 2 μg/ml leupeptin and 1 mM PMSF). After incubation on ice for 10 min, cells were centrifuged at the speed of 6,000 × g at 4℃ for 10 minutes; the cytoplasmic fraction was collected in the supernatant. The pellet was washed with buffer A and then resuspended in buffer B (50 mM Tris, pH 8.0; 120 mM NaCl; 0.5% Nonidet P-40; 1 mM DTT; 2 μg/ml aprotinin; 2 μg/ml leupeptin and 1 mM PMSF). After centrifugation, the nuclear fraction was collected in the supernatant and was assessed by Western blot analysis. The ImageJ software was used to quantify the results of Western blot analysis. All the antibodies used are listed in Table S5. The expression levels of HAT1 and CAF-1 were analyzed by Western blot analysis in liver tissues of human liver-chimeric mice and HBV infected human liver-chimeric mice. The acetylation levels of histone H3K27, H4K5 and H4K12 were measured by Western blot analysis in liver tissues of the mice. Institute Research Ethics Committee at the Nankai University approved the study protocol.

### Statistical Analysis

Statistical significance was assessed by comparing mean values (± SD) using a Student's *t* test for independent groups and was assumed for *P<0.05; **P<0.01; ***P<0.001. Mean ± SD of at least three experiments are shown, in which each experiment was designed by three replicates. One-way analysis of variance was performed to compare HAT1 expression in all individual HBV negative hepatoma cell lines with HBV positive hepatoma cell lines. Pearson's correlation coefficient was used to determine the correlation between HAT1 and HBx mRNA/pgRNA levels in liver tissues of patient.

## Supplementary Material

Supplementary figures and tables.
