# Supplementary material for: HAT1 signaling confers to assembly and epigenetic regulation of HBV cccDNA minichromosome
Source: Theranostics. 2019 Sep 25;9(24):7345–58. doi: 10.7150/thno.37173 (PMC6831306; doi:10.7150/thno.37173)
Supplement: Supplementary file 1 — Supplementary figures and tables. [file thnov09p7345s1.pdf]

Supplementary materials and methods

Plasmid construct

The CDS region of H3, K27 mutant (K to R) H3, H4, K5 mutant (K to R) H4, K12 mutant (K to R) H4 and K5+K12 mutant (K to R) H4 were synthesized and constructed into the plasmid of pCMV-3Tag-1A by Genscript (Nanjing, P.R. China). And this constructed plasmid was named as flag-H3, flag-H3K27mut, flag-H4, flag-H4K5mut, flag-H4K12mut and flag-H4K5K12mut. The plasmids, such as pcDNA3.1-p300, pcDNA3.1-HBX, pSi-HBX and PCH9/3091, were used in our previous studies. The 5'-flanking region (nucleotides -1473/+23) of HAT1 was amplified by PCR from the genomic DNA of HepG2 using specific primers and was cloned into the upstream of the pGL3-Basic vector (Promega, Madison, WI, USA). The resulting plasmid was sequenced and named pGL3-P1. To construct various length luciferase reporter plasmids of HAT1, the regions (-1251/+23, -1029/+23, -705/+23, -432/+23, -183/+23) of HAT1 were amplified by PCR from the pGL3-P1 and were inserted into the pGL3-Basic vector to generate pGL3-P2, pGL3-P3, pGL3-P4, pGL3-P5, pGL3-P6, respectively. The mutant construct of pGL3-P6, termed as pGL3-P6 MUT, which carried a substitution of series nucleotides (position, -21 to -14, such as ATAGTCTA instead of wild type GGGCGGGG) within the binding site of Sp1, was synthesized by Augct (Beijing, China), and then was cloned into the pGL3-Basic vector. The CDS region of HAT1 was amplified by PCR from the cDNA of HepG2.2.15 cells using specific primers and was cloned into the pcDNA3.1 and pGEX-4T-1 vector. The CDS region of HBc was amplified by PCR from the cDNA of HepG2.2.15 cells using specific primers and was cloned into the pCMV-Tag2B and pET-28a vector. All primers are listed in Table S3.

Immunoprecipitation assays

Co-immunoprecipitations were performed using Pierce Co-Immunoprecipitation Kit (Fisher Scientific - Germany, Schwerte, Germany) according to the manufacturer's instructions [1]. Chromatin immunoprecipitation was performed using Pierce Agarose ChIP Kit (#26156, Fisher Scientific) or ChIP kit #ab500 (Abcam, Cambridge, UK).

siRNA and antibodies

The siRNAs used in this study were purchased from RiboBio (Guangzhou, China). All oligonucleotide sequences were listed in Table S4. For Western blot analysis, immunostaining, immunoprecipitation and HBV cccDNA ChIP, the antibodies used in this study were listed in Table S5.

RNA pull-down assays

Biotin-labeled RNA was transcribed *in vitro* using the MEGAscript T7 Kit with biotin-UTP and purified by MEGAclear Kit according to manufacturer's instructions, and then incubated with whole cell lysates. Biotin-labeled transcripts and coprecipitated proteins were isolated with streptavidin beads and then subjected to SDS–PAGE analysis, and further visualized by immunoblotting assay [2].

References

1. Lee HW, Kyung T, Yoo J, Kim T, Chung C, Ryu JY, et al. Real-time single-molecule co-immunoprecipitation analyses reveal cancer-specific Ras signalling dynamics. Nat Commun. 2013; 4: 1505.

2. Li D, Liu X, Zhou J, Hu J, Zhang D, Liu J, et al. Long noncoding RNA HULC

modulates the phosphorylation of YB-1 through serving as a scaffold of extracellular signal-regulated kinase and YB-1 to enhance hepatocarcinogenesis. Hepatology 2017; 65: 1612-1627.

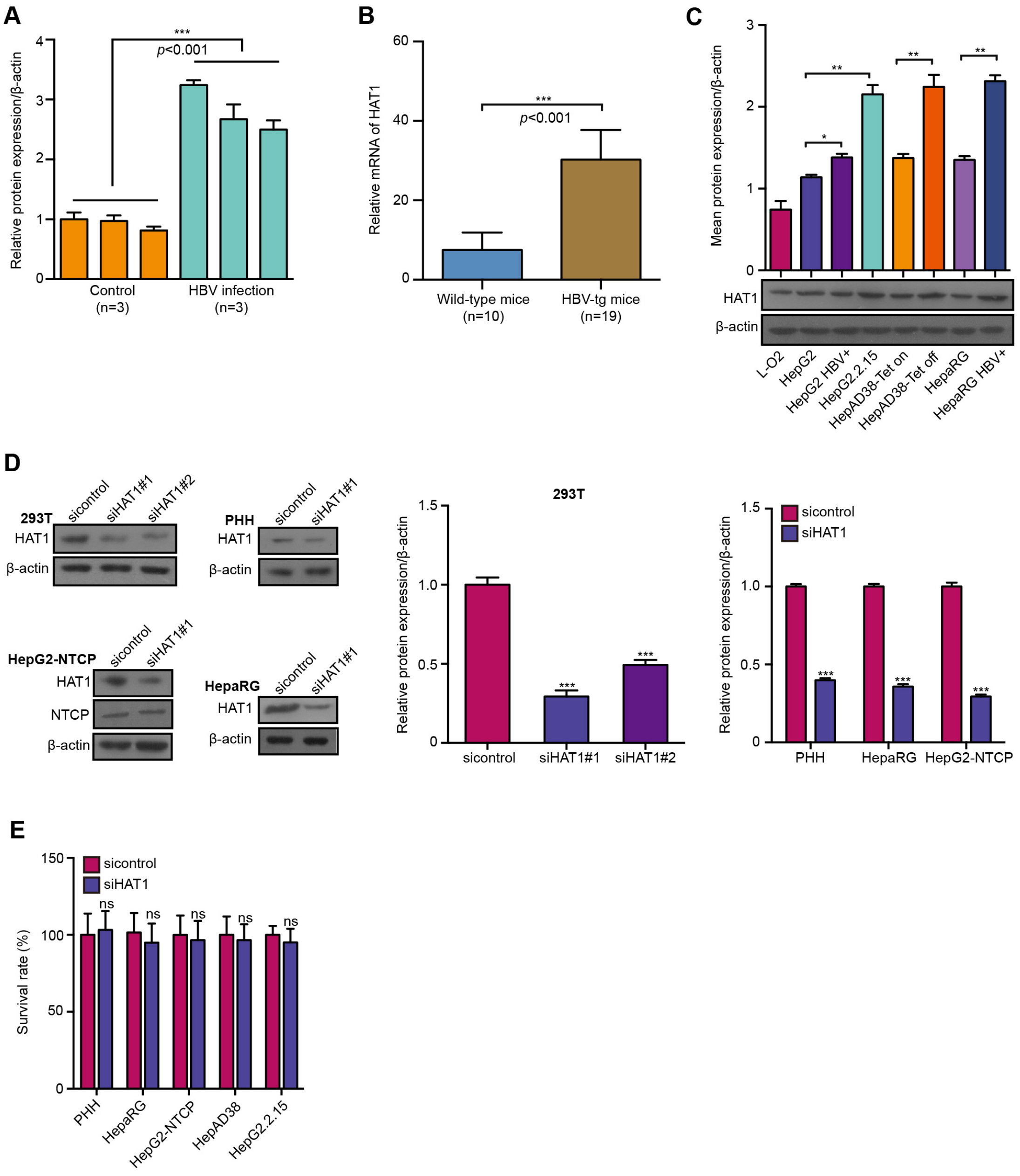

Figure S1. HAT1 is elevated in HBV transgenetic mice, HBV-infected cells and HBV-positive cells. (A) The quantification of Western blot analysis results in Figure 1C was shown. (B) Relative mRNA levels of HAT1 were detected by RT-qPCR in liver tissues from HBV transgenetic mice (HBV-tg mice, n=19) and wild-type mice (n=10). (C) Examination of protein levels of HAT1 in HBV free or HBV-infected and HBV-positive hepatoma cells with the quantitative data of respective Western blot analysis. (D) HEK293T cells were transfected with siHAT1#1 or siHAT1#2 to choose a more effective siRNA of HAT1 by Western blot

analysis. The verification of siHAT1#1 efficiency was performed in the indicated cells, which was used in the following experiment. The expression of NTCP was validated in HepG2-NTCP cells. The cells were transfected with siRNA of HAT1 (100 nM). The quantification of Western blot analysis results was shown. (E) Viability of indicated cells was assessed by MTS assay. The PHH, dHepaRG and HepG2-NTCP cells were infected at MOI of 600 vp/cell with HBV and were continuously transfected with siRNA of HAT1 at -4, 0, 4 and 8 dpi. 12 days after infection, MTS assay was performed. Mean ± SD of at least three experiments are shown, in which each experiment was designed by three replicates. Statistical significant differences are indicated: *P<0.05; **P<0.01; ***P<0.001; ns, no significance.

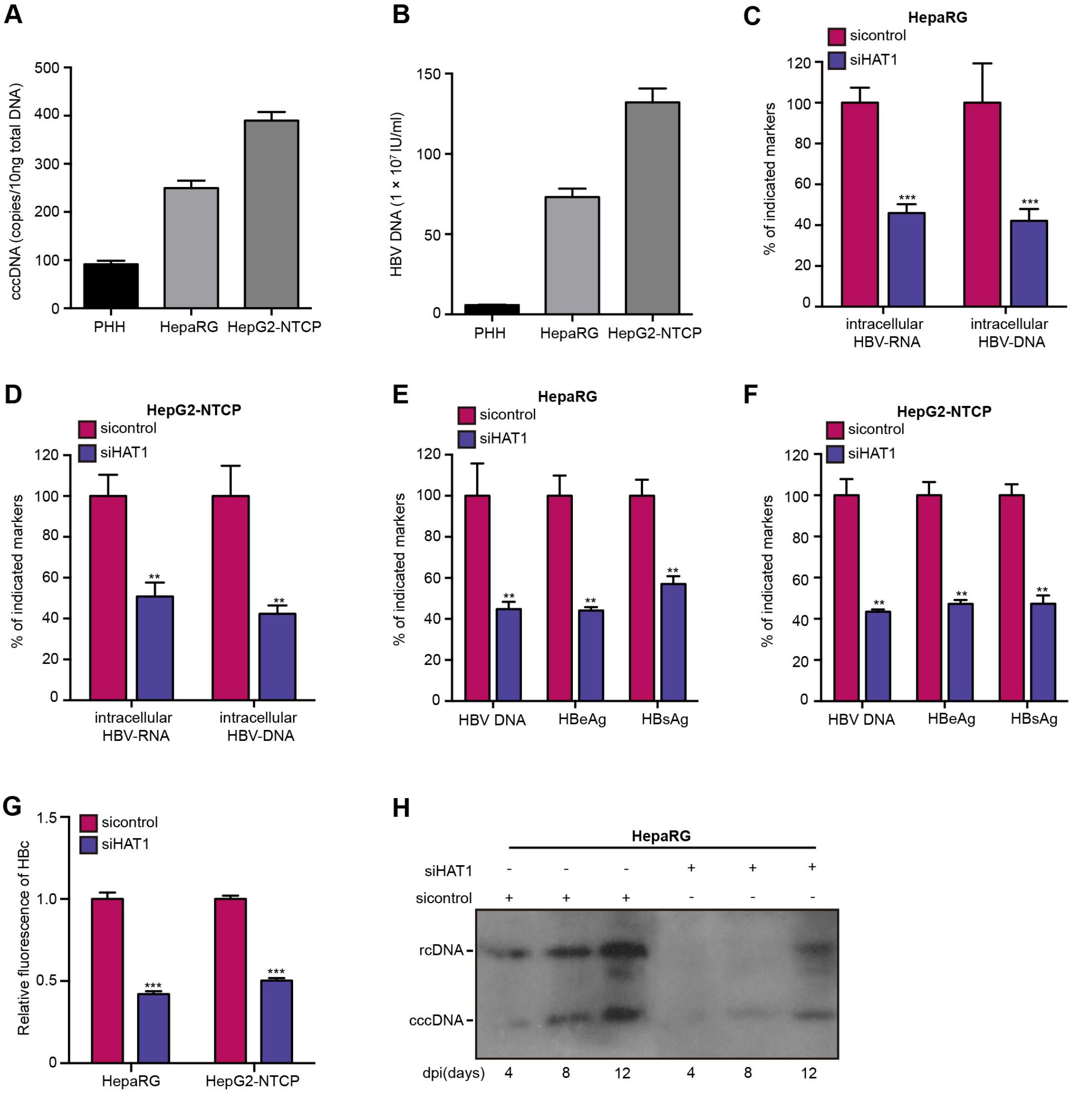

Figure S2. HAT1 contributes to HBV replication and cccDNA accumulation. (A and B) The PHH, dHepaRG and HepG2-NTCP cells were infected with HBV at MOI of 600 vp/cell. The levels of HBV cccDNA in the cells and HBV DNA in the medium were measured by qPCR, respectively. (C-F) The dHepaRG and HepG2-NTCP cells were infected at MOI of 600 vp/cell with HBV and were continuously transfected with siRNA of HAT1 at -4, 0, and 4 dpi (days post-infection). (C and D) The intracellular HBV-RNA and intracellular HBV-DNA were detected 8 dpi by qPCR in the cells. (E and F) The levels of HBV DNA, HBeAg and HBsAg in the medium were measured by qPCR and ELISA in the cells. (G) The

quantification of HBc fluorescence in Figure 1F was analyzed by ImageJ software. (H) The dHepaRG cells were infected with HBV at MOI of 600 vp/cell and were continuously transfected with siHAT1 (100 nM) at -4, 0,and 4 dpi. HBV cccDNA was analyzed 8 dpi by Southern blot analysis in the dHepaRG cells. The EcoRI digested-DNA and EcoRI undigested-DNA were examined to verify the cccDNA and rcDNA in the cells. Mean ± SD of at least three experiments are shown, in which each experiment was designed by three replicates. Statistical significant differences are indicated: ***P*<0.01; ****P*<0.001.

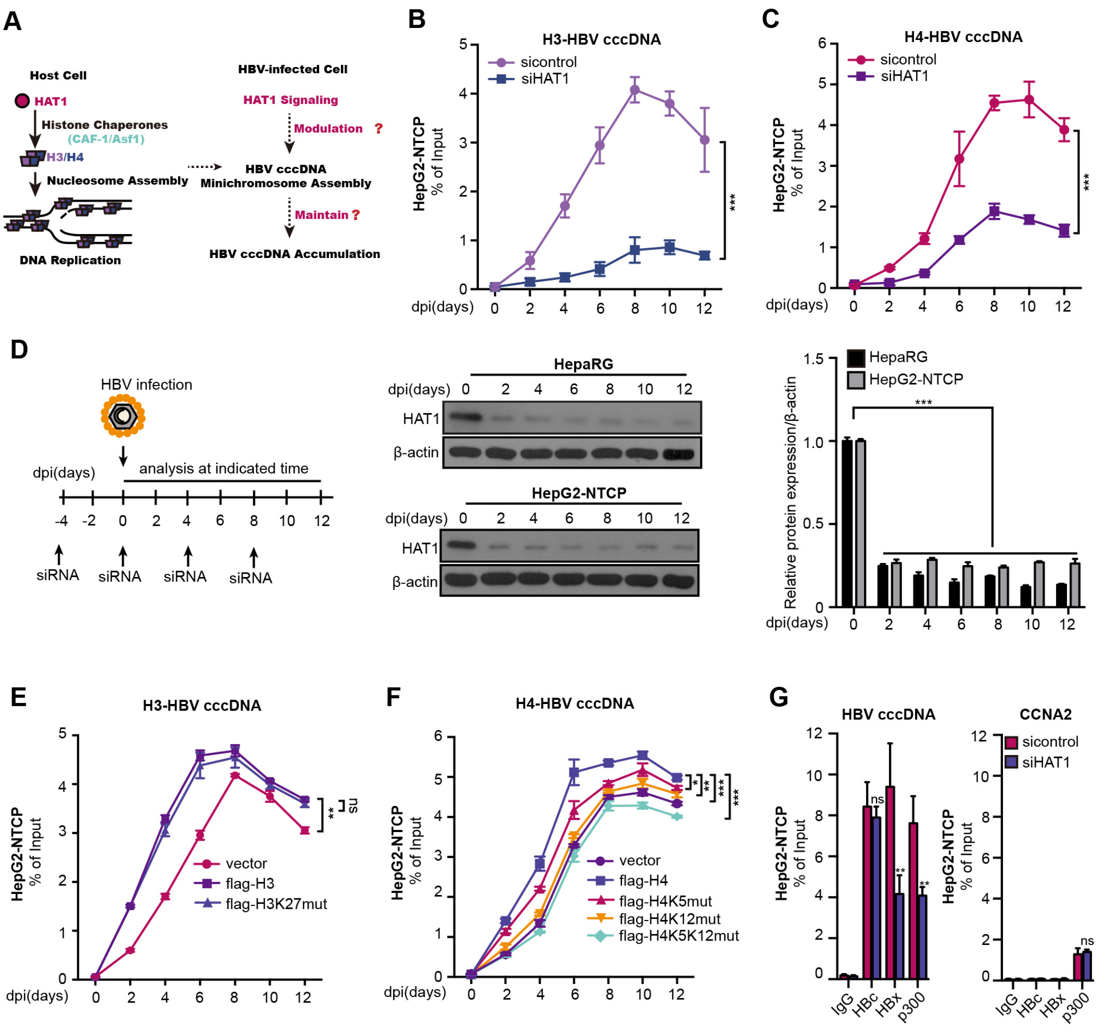

Figure S3. HAT1 confers to the assembly of HBV cccDNA minichromosome. (A) The hypothesis for the function of HAT1 in modulation of HBV cccDNA minichromosome was

shown in a min-model. See text for description. (B and C) The assembly of histone H3/H4 onto HBV cccDNA was examined by ChIP-qPCR at 0, 2, 4, 6, 8, 10 and 12 dpi in the HepG2-NTCP cells. (D) A model of flow chart for the experience, testing the effect of siHAT1 on assembly of histone H3/H4 onto HBV cccDNA (left panel). The dHepaRG and HepG2-NTCP cells were infected at MOI of 600 vp/cell with HBV and were continuously transfected with siRNA of HAT1 (100 nM) at -4, 0, 4 and 8 dpi. The siHAT1 efficiency was confirmed by Western blot analysis at indicated dpi in the cells (middle panel). The quantification of Western blot analysis results was shown (right panel). (E) The assembly of histone H3 onto cccDNA was examined by ChIP-qPCR at indicated dpi in HBV-infected HepG2-NTCP cells continuously transfected with flag-vector/flag-H3/flag-H3K27mut at -4, 0, 4 and 8 dpi. (F) The assembly of histone H4 onto cccDNA was examined by ChIP-qPCR at indicated dpi in HBV-infected HepG2-NTCP cells continuously transfected with flag-vector/flag-H4/flag-H4K5mut/ flag-H4K12mut/flag-H4K5K12mut at -4, 0, 4 and 8 dpi, respectively. (G) The deposition of HBc, HBx and p300 onto cccDNA minichromosome or the promoter of CCNA2 was verified 8 dpi by ChIP-qPCR in HBV-infected HepG2-NTCP cells transfected with siHAT1. Mean ± SD of at least three experiments are shown, in which each experiment was designed by three replicates. Statistical significant differences are indicated: *$P<0.05$; **$P<0.01$; ***$P<0.001$; ns, no significance.

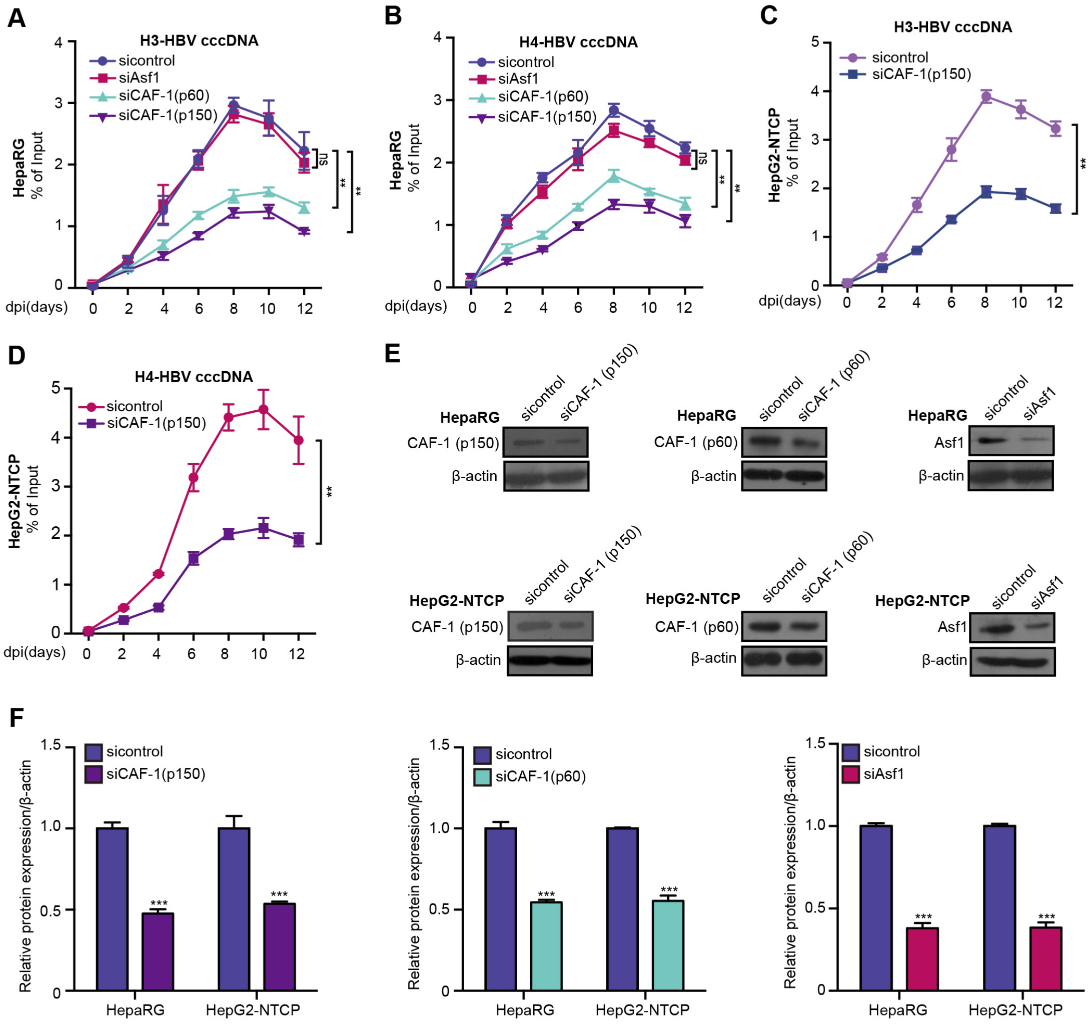
A
H3-HBV cccDNA
HepaRG % of Input
sicontrol
siAsf1
siCAF-1(p60)
siCAF-1(p150)
ns
**
**
dpi(days)
B
H4-HBV cccDNA
HepaRG % of Input
sicontrol
siAsf1
siCAF-1(p60)
siCAF-1(p150)
ns
**
**
dpi(days)
C
H3-HBV cccDNA
HepG2-NTCP % of Input
sicontrol
siCAF-1(p150)
**
dpi(days)
D
H4-HBV cccDNA
HepG2-NTCP % of Input
sicontrol
siCAF-1(p150)
**
dpi(days)
E
HepaRG
sicontrol
siCAF-1 (p150)
CAF-1 (p150)
β-actin
HepaRG
sicontrol
siCAF-1 (p60)
CAF-1 (p60)
β-actin
HepaRG
sicontrol
siAsf1
Asf1
β-actin
HepG2-NTCP
sicontrol
siCAF-1 (p150)
CAF-1 (p150)
β-actin
HepG2-NTCP
sicontrol
siCAF-1 (p60)
CAF-1 (p60)
β-actin
HepG2-NTCP
sicontrol
siAsf1
Asf1
β-actin
F
Relative protein expression/β-actin
sicontrol
siCAF-1(p150)
***
***
HepaRG
HepG2-NTCP
Relative protein expression/β-actin
sicontrol
siCAF-1(p60)
***
***
HepaRG
HepG2-NTCP
Relative protein expression/β-actin
sicontrol
siAsf1
***
***
HepaRG
HepG2-NTCP

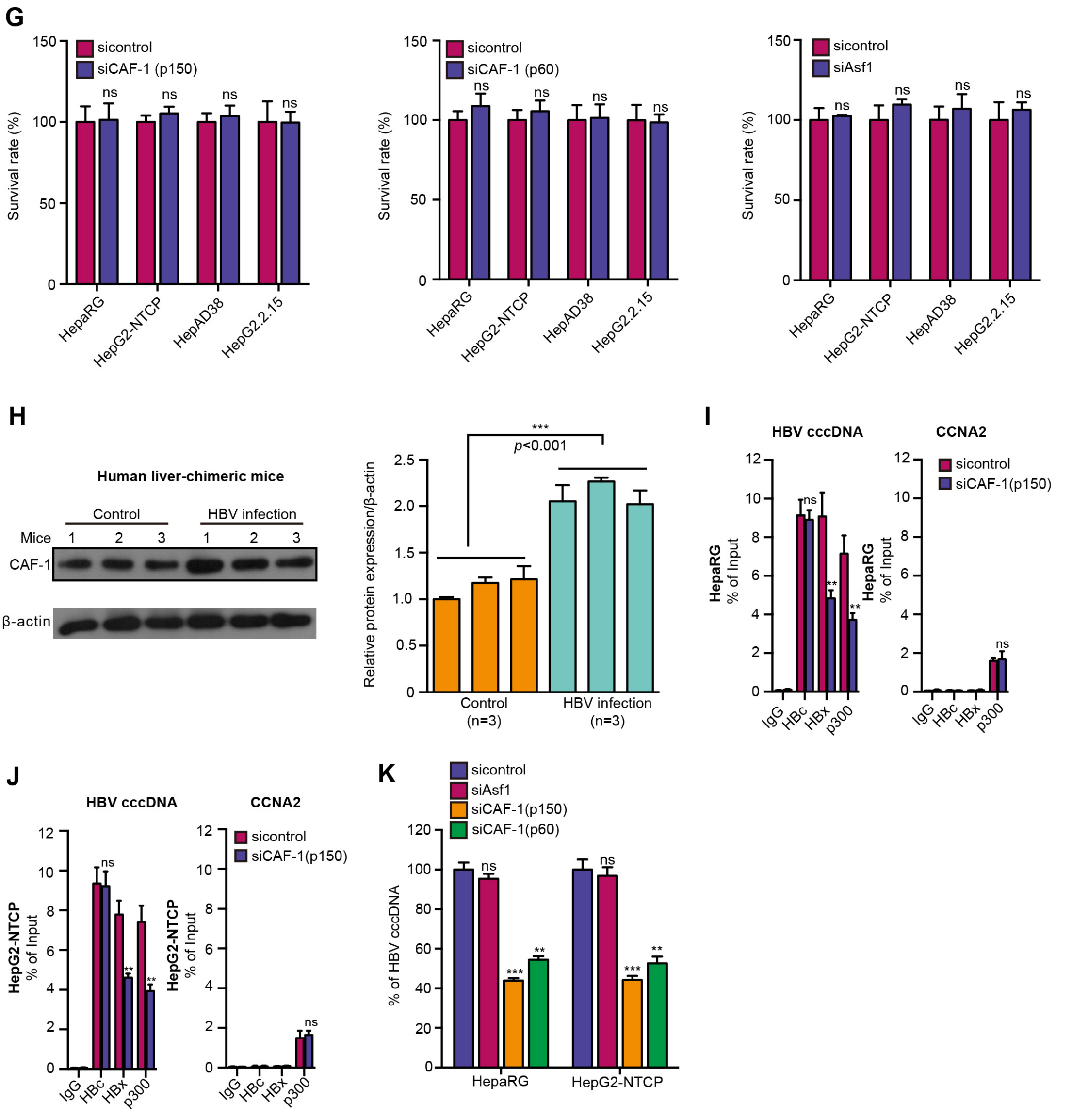

Figure S4. HAT1/CAF-1 signaling confers to the assembly of HBV cccDNA minichromosome. (A and B) The dHepaRG cells were infected at MOI of 600 vp/cell with HBV and were continuously transfected with siCAF-1 (p150), siCAF-1 (p60) or siAsf1 at -4, 0, 4 and 8 dpi. The assembly of histone H3/H4 onto HBV cccDNA was examined by ChIP-qPCR at 0, 2, 4, 6, 8, 10 and 12 dpi in the cells. (C and D) The assembly of histone H3/H4 onto HBV cccDNA was determined by ChIP-qPCR at 0, 2, 4, 6, 8, 10 and 12 dpi in the HepG2-NTCP cells transfected with siCAF-1 (p150). (E and F) The verification of siCAF-1 (p150), siCAF-1 (p60) and siAsf1 efficiency was performed by Western blot

analysis in the indicated cells. The quantification of Western blot analysis results was shown (CAF-1 (p150), left panel; CAF-1 (p60), middle panel; Asf1, right panel). (G) Viability of indicated cells was assessed by MTS assay. The dHepaRG and HepG2-NTCP cells were infected at MOI of 600 vp/cell with HBV and were continuously transfected with siCAF-1 (p150), siCAF-1 (p60) or siAsf1 at -4, 0, 4 and 8 dpi. 12 days after infection, MTS assay was performed. (H) The protein levels of CAF-1 were examined in human liver-chimeric mice (n=3) and HBV-infected human liver-chimeric mice (n=3) by Western blot analysis. The quantification of Western blot analysis results was shown. (I and J) The deposition of HBc, HBx and p300 onto HBV cccDNA minichromosome or the promoter of CCNA2 was verified 8 dpi by ChIP-qPCR in the dHepaRG and HepG2-NTCP cells transfected with siCAF-1 (p150). (K) HBV cccDNA was analyzed by selective qPCR in HBV-infected dHepaRG and HepG2-NTCP cells transfected with 100 nM siCAF-1 (p150), siCAF-1 (p60) or siAsf1. Mean ± SD of at least three experiments are shown, in which each experiment was designed by three replicates. Statistical significant differences are indicated: **P<0.01; ***P<0.001; ns, no significance.

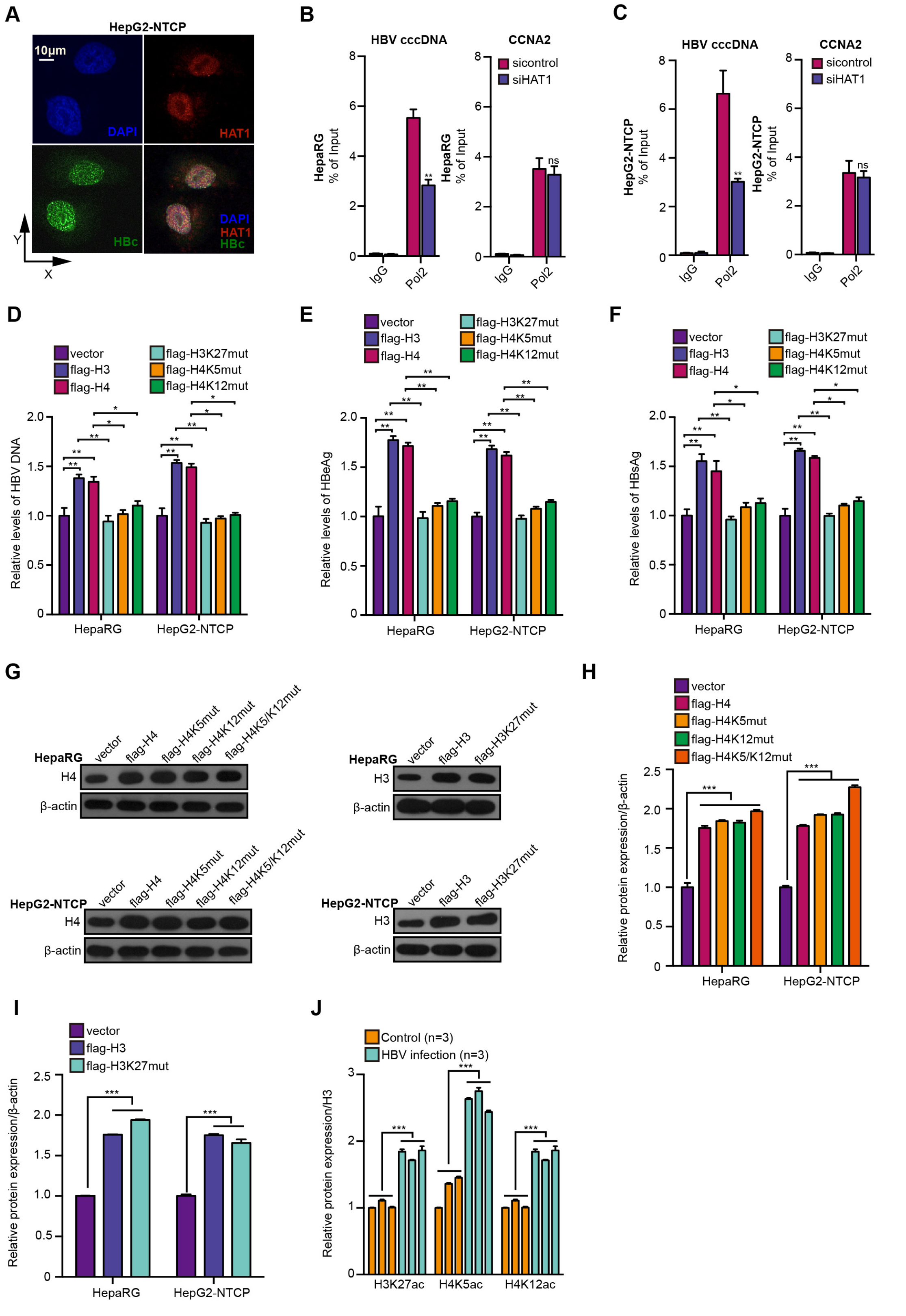

Figure S5. HAT1 promotes histone acetylation on HBV cccDNA minichromosome. (A) The colocalization of HAT1 and HBc in the cells was analyzed 8 dpi by confocal microscopy in

the HepG2-NTCP cells. (B and C) The binding of Pol2 on cccDNA was assessed 8 dpi by ChIP-qPCR in the HBV-infected dHepaRG and HepG2-NTCP cells transfected with siHAT1. (D-I) The dHepaRG and HepG2-NTCP cells were infected at MOI of 600 vp/cell with HBV. The levels of HBV DNA, HBeAg and HBsAg in the medium were examined by qPCR and ELISA in the cells continuously transfected with flag-vector/flag-H3/flag-H4/flag-H3K27mut/flag-H4K5mut/flag-H4K12mut at -4, 0, 4 and 8 dpi, respectively. The efficiency of overexpression of histone H3 and H4 was validated by Western blot analysis in the cells. The quantification of Western blot analysis results of Figure S5G was shown in Figure S5H and I. (J) The quantification of Western blot analysis results of Figure 3H was shown. Mean ± SD of at least three experiments are shown, in which each experiment was designed by three replicates. Statistical significant differences are indicated: *$P<0.05$; **$P<0.01$; ns, no significance.

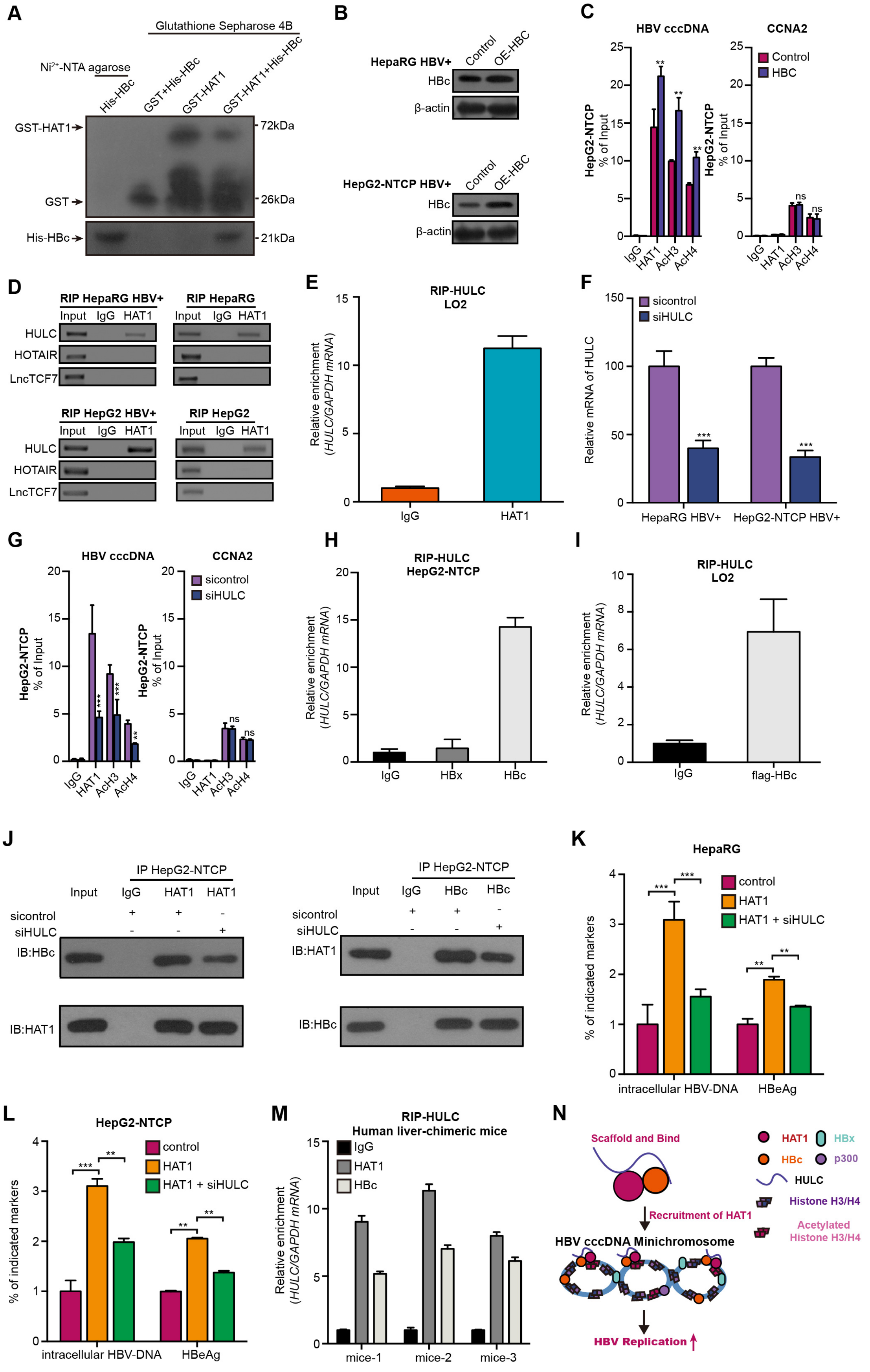

Figure S6. HAT1 is recruited to cccDNA minichromosome by lncRNA HULC-scaffold HBc.

(A) The combination of HAT1 and HBc was assessed by GST pull-down assays. (B) The efficiency of HBc overexpression was determined 8 dpi by Western blot analysis in the HBV-infected dHepaRG and HepG2-NTCP cells transfected with pcDNA3.1-HBC (2 μg/well). OE-HBC: overexpression of HBC. (C) The deposition of HAT1 and acetylation of histone including AcH3 and AcH4 on cccDNA minichromosome or the promoter of CCNA2 were verified 8 dpi by ChIP-qPCR in HBV-infected HepG2-NTCP cells continuously transfected with pcDNA 3.1-HBC (2 μg/well). (D and E) The interaction of indicated lncRNAs with HAT1 was examined by RIP-PCR assays in the indicated cells. (F) The efficiency of or siHULC was determined 8 dpi by RIP-qPCR assays in the HBV-infected dHepaRG and HepG2-NTCP cells transfected with siHULC (100 nM). (G) The deposition of HAT1 and acetylation of histone including AcH3 and AcH4 on cccDNA minichromosome or the promoter of CCNA2 were analyzed 8 dpi by ChIP-qPCR in HBV-infected HepG2-NTCP cells continuously transfected with siHULC (100 nM). (H) The combination of HULC with HBc or HBx was examined 8 dpi by RIP-qPCR assays in HBV-infected HepG2-NTCP cells. (I) The combination of HULC with HBc was analyzed by RIP-qPCR assays in LO2 cells transfected with pCMV-HBC. (J) The combination of HAT1 and HBc was analyzed 8 dpi by immunoprecipitation assays in HBV-infected HepG2-NTCP cells continuously transfected with siHULC (100 nM). (K and L) The intracellular HBV-DNA and HBeAg were determined 8 dpi by qPCR and ELISA in HBV-infected dHepaRG and HepG2-NTCP cells continuously transfected with pcDNA3.1-HAT1 (2 μg/well) or cotransfected with pcDNA3.1-HAT1 (2 μg/well) and siHULC (100 nM) at -4, 0, and 4 dpi. (M) The interaction of HULC with HAT1 and HBc was analyzed by RIP-qPCR assays in the liver of HBV-infected human

liver-chimeric mice (n=3). (N) A model for role of HULC-scaffold HAT1/HULC/HBc complex in histone acetylation of cccDNA minichromosome. Mean ± SD of at least three experiments are shown, in which each experiment was designed by three replicates. Statistical significant differences are indicated: ***P*<0.01; ****P*<0.001; ns, no significance.

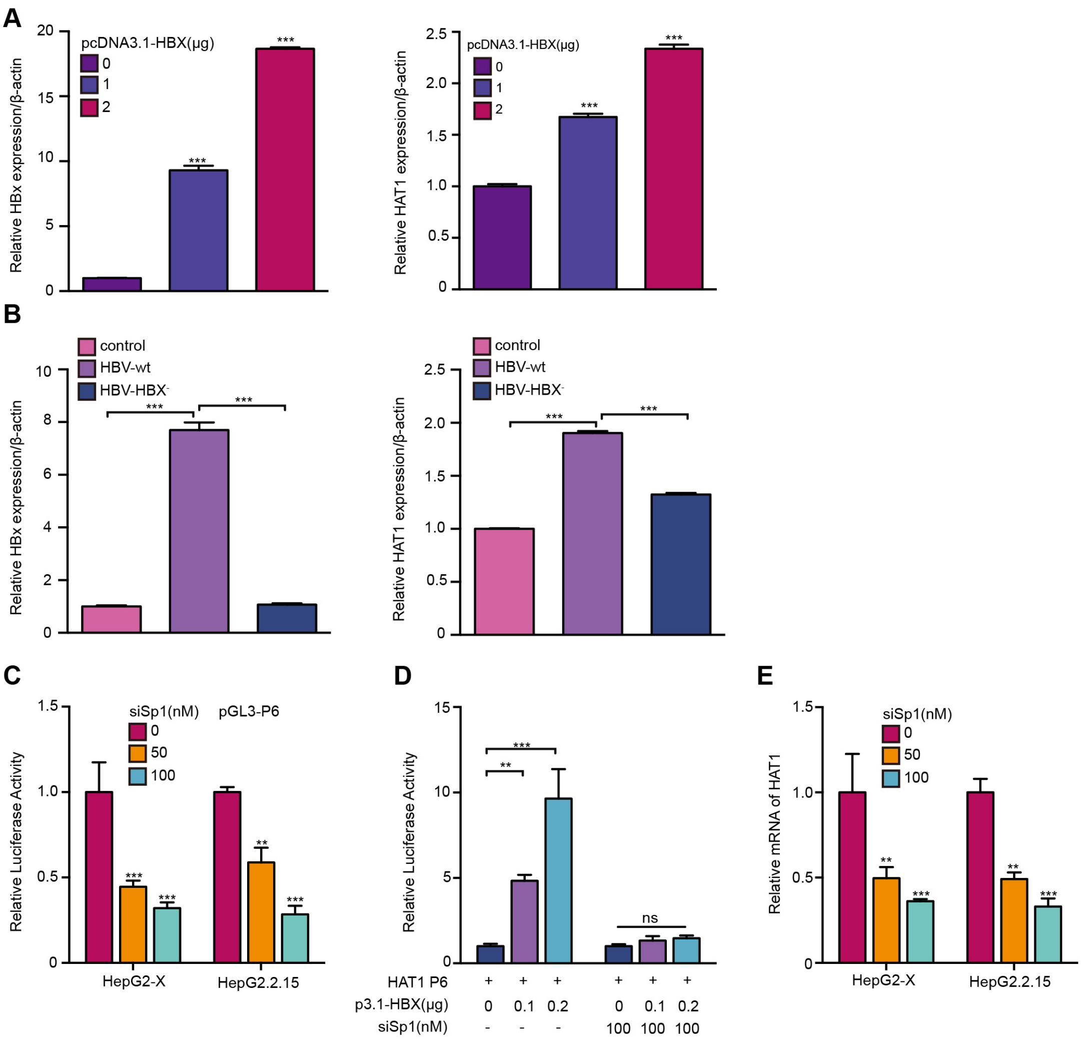

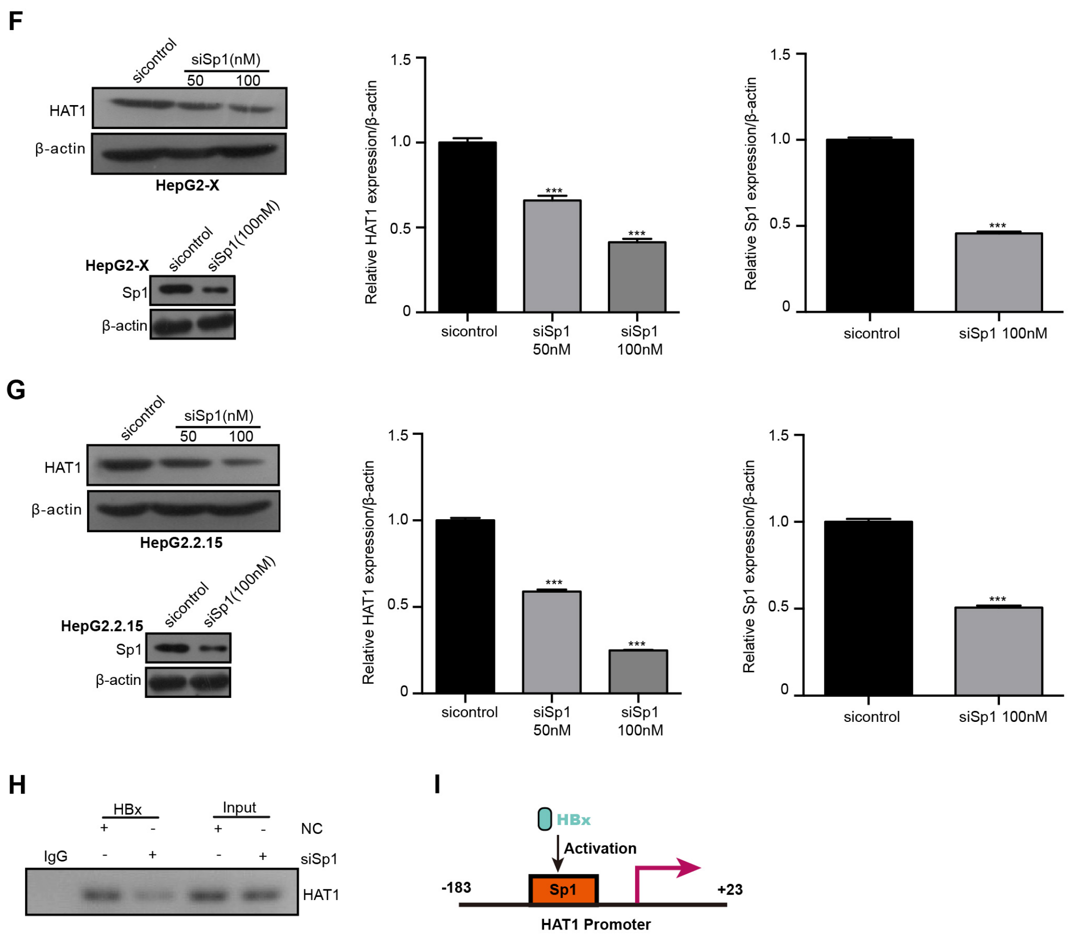

Figure S7. HBV stimulates HAT1 promoter through HBx-co-activated transcriptional factor Sp1. (A and B) The quantification of Western blot analysis results in Figure 5B and C was shown, respectively. (C) Luciferase activities of HAT1 P6 promoter were measured in HepG2-X and HepG2.2.15 cells transfected with siSp1. (D) Luciferase activities of HAT1 P6 promoter were determined in HepG2 cells transfected with pcDNA3.1-HBX or cotransfected with pcDNA3.1-HBX and siSp1. (E-G) The expression of HAT1 was measured by RT-qPCR and Western blot analysis in HepG2-X and HepG2.2.15 cells transfected with siSp1. The quantification of Western blot analysis results was shown. (H) ChIP assays were performed to confirm the interaction of HBx with the promoter region of HAT1. (I) A model for the function of HBx in up-regulating HAT1 by activating HAT1 promoter in an Sp1-dependent manner was shown. Mean ± SD of at least three experiments are shown, in which each

experiment was designed by three replicates. Statistical significant differences are indicated: **P<0.01; ***P<0.001; ns, no significance.

Table S1. Information of human liver-chimeric mice

Group	Mouse (No.)	Albumin (μg/mL)	HBV DNA (IU/mL)	cccDNA (copies/10ng total DNA)
Control	10.16-14	1916.18	—	—
	10.16-15	2065.83	—	—
	10.16-19	3242.68	—	—
HBV infection	7.25-13	1093.72	7.81E+05	83
	7.25-15	1571.23	4.20E+06	95
	8.10-15	630.57	5.82E+06	113

Table S2: The characteristics of non-tumor liver tissues of HCC patients

No.	Age	Gender	Organ	Chronic hepatitis with HBV	HBV DNA	cccDNA
1	59	F	Liver	+	+	+
2	60	M	Liver	+	+	-
3	65	M	Liver	+	+	+
4	43	M	Liver	+	+	+
5	60	F	Liver	+	+	-
6	41	M	Liver	+	+	+
7	45	M	Liver	+	+	+
8	56	M	Liver	+	+	-
9	70	M	Liver	+	+	-
10	67	M	Liver	+	+	+
11	59	M	Liver	+	+	+
12	57	M	Liver	+	+	+
13	61	F	Liver	+	+	-
14	51	F	Liver	+	+	+
15	56	M	Liver	+	+	+
16	60	M	Liver	+	+	-
17	54	M	Liver	+	+	+
18	36	M	Liver	+	+	-
19	46	M	Liver	+	+	+
20	60	M	Liver	+	+	+
21	59	M	Liver	+	+	-
22	57	F	Liver	+	+	+
23	51	M	Liver	+	+	-
24	38	M	Liver	+	+	+
25	56	M	Liver	+	+	-
26	49	M	Liver	+	+	+
27	58	F	Liver	+	+	-
28	46	M	Liver	+	+	+
29	56	M	Liver	+	+	+
30	54	M	Liver	+	+	-
31	39	M	Liver	+	+	-
32	53	M	Liver	+	+	+

33	60	M	Liver	+	+	+
34	66	M	Liver	+	+	-
35	60	F	Liver	+	+	+
36	59	M	Liver	+	+	-
37	69	M	Liver	+	+	+
38	38	M	Liver	+	+	+
39	56	M	Liver	+	+	+
40	53	F	Liver	-	-	-
41	47	M	Liver	+	-	-
42	67	M	Liver	-	-	-
43	49	M	Liver	-	-	-

Table S3: Primer list (for HBV primers the positions are indicated relative to EcoRI site)

No.	Gene	Primer	Sequence(5′-3′)	Position
**PCR**				
1	HBV cccDNA	forward	GCCTATTGATTGGAAAGTATGT	969-990
		reverse	AGCTGAGGCGGTATCTA	1992-2008
2	hCCNA2- CHIP	forward	CCTGCTCAGTTTCCTTTGGT	NG_052974.1: 4982-5001
		reverse	AGACGCCCAGAGATGCAG	NG_052974.1: 5132-5149
3	HAT1 mRNA	forward	GAAATGGCGGGATTTGGTGC	NM_003642.4: 38-57
		reverse	TGTAGCCTACGGTCGCAAAG	NM_003642.4: 685-704
4	HAT1 mouse	forward	ACACCAACACAGCAATCG	NM_026115.4: 112-129
		reverse	CATCTGCCTCCACACAATC	NM_026115.4: 327-345
5	GAPDH	forward	AACGGATTTGGTCGTATTG	NM_002046.7: 101-119
		reverse	GGAAGATGGTGATGGGATT	NM_002046.7: 290-308
6	GAPDH mouse	forward	CCTGCCAAGTATGATGACAT	NM_001289726.1: 840-859
		reverse	GTTGCTGTAGCCGTATTCA	NM_001289726.1: 1034-1052
7	pgRNA	forward	CTCCTCCAGCTTATAGACC	2283-2301
		reverse	GTGAGTGGGCCTACAAA	2589-2605
8	rcDNA	forward	GGAGGGATACATAGAGGTTCCTTGA	409-433
		reverse	GTTGCCCGTTTGTCCTCTAATTC	334-356
9	HULC	forward	ATCTGCAAGCCAGGAAGAGTC	NR_004855.2: 96-116
		reverse	CTTGCTTGATGCTTTGGTCTGT	NR_004855.2: 258-279
10	HOTAIR	forward	CAAACAGAGTCCGTTCAGTGTC	NR_047517.1: 1286-1307
		reverse	GTGGATTCCTGGGTGGGT	NR_047517.1: 1620-1637
11	LncTCF7	forward	AGGAGTCCTTGGACCTGAGC	NR_131252.1: 202-221
		reverse	AGTGGCTGGCATATAACCAACA	NR_131252.1: 296-317
12	HBx	forward	ATGGCTGCTAGGCTGTGC	1374-1391
		reverse	TTAGGCAGAGGGGAAAAAGTTG	1817-1838
**CRISPR-Cas9**				
13	HAT1-sgRNA-1	forward	GACCGTAGGCTGACATGACATGTAG	
		reverse	AAACCTACATGTCATGTCAGCCTAC	
14	HAT1-sgRNA-2	forward	GACCGGCTACGCTCTTTGCGACCGT	
		reverse	AAACACGGTCGCAAAGAGCGTAGCC	
**Plasmid construction**				
15	PGL3-P1	forward	GGGGTACCCCGGGAGGCGGAGGCTG	
		reverse	CCCTCGAGCCCGGGCCGGAAGTGAC	
16	PGL3-P2	forward	GGGGTACCTGCATACTGAGAATGAAC	
		reverse	CCCTCGAGCCCGGGCCGGAAGTGAC	

17	PGL3-P3	forward	GGGGTACCTCCTCTGGGGAAAGCAG
		reverse	CCCTCGAGCCCGGGCCGGAAGTGAC
18	PGL3-P4	forward	GGGGTACCTAGTAGAGACGGGGTTTC
		reverse	CCCTCGAGCCCGGGCCGGAAGTGAC
19	PGL3-P5	forward	GGGGTACCAAGTGAAAGCTTCTCCCG
		reverse	CCCTCGAGCCCGGGCCGGAAGTGAC
20	PGL3-P6	forward	GGGGTACCGCCTCCTTCTCCACTT
		reverse	CCCTCGAGCCCGGGCCGGAAGTGAC
21	PGL3-P6 MUT	forward	GGGGTACCGCCTCCTTCTCCACTT
		reverse	CCCTCGAGATATCAGAGGGATACAG
22	pcDNA3.1-HAT1	forward	CCGGAATTCATGGCGGGATTTGGTGCTATGG
		reverse	CCGCTCGAGTTACTCTTGAGCAAGTCGTTCA
23	GST-HAT1	forward	CCGGAATTCATGGCGGGATTTGGTGCTATGG
		reverse	CCGCTCGAGTTACTCTTGAGCAAGTCGTTCA
24	His-HBc	forward	CGCGGATCCATGGACATCGACCCTTATAAAGA
		reverse	CCGCTCGAGCATTGAGATTCCCGAGAT

Table S4: target sequences of siRNAs

siRNAs	Target sequence (5′-3′)
siHAT1 #1	GCUACAGACUGGAUAUUAA
siHAT1 #2	GAAAGAUGGCACUACUUUC
siCAF-1 (p150)	AAGGAGAAGGCGGAGAAGCAG
siCAF-1 (p60)	AAGCGUGUGGCUUUCAAUGUU
siAsf1	AAUCCAGGACUCAUUCCAGAU
siHULC	CCUCCAGAACUGUGAUCC
siSp1	GCCAAUAGCUACUCAACUA

Table S5: antibodies used in this study

Antibodies	Origin
HAT1 Polyclonal Antibody	Proteintech 11432-1-AP
HAT1 Monoclonal Antibody	Abcam ab194296
CAF-1 Monoclonal Antibody	Abcam ab126625
Asf1 Polyclonal Antibody	Proteintech 10784-1-AP
H3 Polyclonal Antibody	Abcam ab1791
H4 Polyclonal Antibody	Abcam ab7311
HBc Monoclonal Antibody	Abcam ab8639
HBx Polyclonal Antibody	Abcam ab39716
NTCP Polyclonal Antibody	Abcam ab131084
p300 Polyclonal Antibody	Abcam ab10485
AcH3 Polyclonal Antibody	Millipore 06-599
AcH4 Polyclonal Antibody	Millipore 06-598
H3K27ac Polyclonal Antibody	Abcam ab4729
H4K5ac Monoclonal Antibody	Abcam ab51997
H4K12ac Polyclonal Antibody	Abcam ab46983
RNA Polymerase 2 Monoclonal Antibody	Abcam ab817
GST tag Monoclonal Antibody	Proteintech 66001-1-Ig
His-Tag Monoclonal Antibody	Proteintech 66005-1-Ig
β-actin Monoclonal Antibody	Sigma-Aldrich A2228
